# The Comparison of Human and Machine Performance in Object Recognition

**DOI:** 10.3390/bs16010109

**Published:** 2026-01-13

**Authors:** Gokcek Kul, Andy J. Wills

**Affiliations:** School of Psychology, Faculty of Health, University of Plymouth, Plymouth PL4 8AA, UK; andy.wills@plymouth.ac.uk

**Keywords:** object recognition, human–machine comparison, deep neural networks, comparative psychology

## Abstract

Deep learning models have advanced rapidly, leading to claims that they now match or exceed human performance. However, such claims are often based on closed-set conditions with fixed labels, extensive supervised training, and do not considering differences between the two systems. Recent findings also indicate that some models align more closely with human categorisation behaviour, whereas other studies argue that even highly accurate models diverge from human behaviour. Following principles from comparative psychology and imposing similar constraints on both systems, this study investigates whether these models can achieve human-level accuracy and human-like categorisation through three experiments using subsets of the ObjectNet dataset. Experiment 1 examined performance under varying presentation times and task complexities, showing that while recent models can match or exceed humans under conditions optimised for machines, they struggle to generalise to certain real-world categories without fine-tuning or task-specific zero-shot classification. Experiment 2 tested whether human performance remains stable when shifting from N-way categorisation to a free-naming task, while machine performance declines without fine-tuning; the results supported this prediction. Additional analyses separated detection from classification, showing that object isolation improved performance for both humans and machines. Experiment 3 investigated individual differences in human performance and whether models capture the qualitative ordinal relationships characterising human categorisation behaviour; only the multimodal CoCa model achieved this. These findings clarify the extent to which current models approximate human categorisation behaviour beyond mere accuracy and highlight the importance of incorporating principles from comparative psychology while considering individual differences.

## 1. Introduction

Machine learning is a broad field of artificial intelligence that enables computer algorithms to learn and recognise patterns on a task directly from examples without requiring explicit instructions or rules from a human (Majaj & Pelli, 2018). In recent years, as in other domains of Artificial Intelligence (AI), advances in computer vision have led to discussions about disparities between human and computer perception. In 2012, AlexNet (Krizhevsky et al., 2012) achieved a significant breakthrough by outperforming prior models on an object recognition benchmark. Following this influential work, several engineers and neuroscientists have claimed that DCNNs (Deep Convolutional Neural Networks) can, in some cases, match or exceed human performance on object recognition tasks (Dehghani et al., 2023; He et al., 2015; Krizhevsky et al., 2012; LeCun et al., 2015; Russakovsky et al., 2015; Zhu et al., 2017). However, such claims often rely on top-5 accuracy, inconsistent category definitions, closed-set evaluations, and fail to balance the limitations of human and machine perception (Firestone, 2020).

Performance gaps become clearer when moving from benchmarks that use independent and identically distributed (IID) test sets, such as ImageNet, to more challenging out-of-distribution (OOD) tests. Although ImageNet offers a large-scale variety, it suffers from limitations, including mislabelling (Beyer et al., 2020; Russakovsky et al., 2015), constrained variation in the representation of objects in real-world environments (Russakovsky et al., 2015), and is often associated with particular backgrounds where objects are frequently shown in stereotypical orientations with minimal occlusion (Barbu et al., 2019). These characteristics introduce inherent biases into the dataset, leading models to rely on superficial statistical regularities, thereby reducing generalisation and robustness under OOD conditions (Barbu et al., 2019; Cooper et al., 2021; Geirhos et al., 2019; Taori et al., 2020; Timme et al., 2020)**.**

Although newer architectures such as vision transformers (ViTs) and Contrastive Language Image Pre-Training (CLIP) have shown improved robustness (Geirhos et al., 2021; Kolesnikov et al., 2020), even models trained for synthetic distortions often struggle with real-world environmental variations (Taori et al., 2020), such as pose transformations—cases where the orientation or viewpoint of an object changes significantly (Abbas & Deny, 2023; Alcorn et al., 2019; Madan et al., 2021). Moreover, achieving good performance on challenging, more realistic OOD image sets, which include distortions, occlusions, and perspectives not seen during training, requires extensive retraining or fine-tuning for DNNs to adapt (Geirhos et al., 2018). Even DNNs that were trained to handle known distortions fail to generalise beyond them; humans, by contrast, are particularly good at recognising objects under new and varied conditions, demonstrating high robustness and adaptability to OOD scenarios (Geirhos et al., 2018).

While achieving or exceeding human-level performance remains a key benchmark goal in evaluating machine vision models, researchers now emphasise that it is equally important to understand the differences in the mechanisms of the two systems—beyond mere accuracy comparisons (Firestone, 2020; Geirhos et al., 2020). According to Wichmann and Geirhos (2023), model assessment is inherently multidimensional, involving the examination of responses and error patterns and the evaluation of how closely DNN classification outputs align with human decision-making at a fine-grained level. Some recent studies in computer vision (e.g., Geirhos et al., 2021; Jaini et al., 2023) have suggested that more advanced machine models are narrowing the gap in OOD distortion robustness compared to humans, indicating a better resemblance to human object recognition systems, such as demonstrating improved shape bias (Geirhos et al., 2021; Jaini et al., 2023) and error consistency with human responses (Geirhos et al., 2021). However, other studies indicate that even high-performing models often diverge from human-like categorisation behaviour (Bowers et al., 2023; Dehghani et al., 2023), and even claim that this problem is worsening with the most advanced DNNs, despite their high accuracy (Fel et al., 2022).

**Figure 1:**
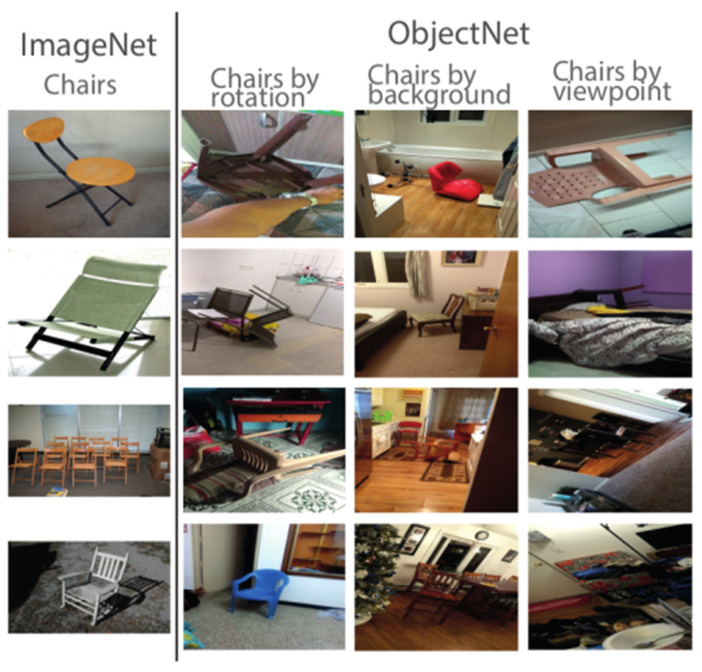
Example pictures from ImageNet (**left column**) and ObjectNet (**right column**). In ObjectNet, objects are photographed from a broader range of orientations and viewpoints against a broader range of backgrounds. This figure is a modified version of Figure 2 from Barbu et al. (2019).

Of central relevance to the current study, there have been attempts to evaluate the performance of DNNs on OOD image test sets considered to be better controlled and more representative of everyday-life scenes than benchmark IID datasets. One notable example is ObjectNet (Barbu et al., 2019), a dataset designed to test recognition across varied viewpoints, backgrounds, and orientations (see Figure 1), containing 50,000 real-world images in 313 categories, with 113 of those overlapping with ImageNet. ObjectNet reveals substantial performance drops in DCNNs: from around 70% top-1 accuracy on ImageNet to roughly 30% on ObjectNet—an approximately 40–45% decrease for overlapping categories. While this decline reflects the dataset’s increased difficulty, the benchmark has been criticised for conflating two related but distinct tasks: object recognition(identifying objects in images) and object detection (locating and identifying multiple objects in cluttered scenes) (Borji, 2021). However, typical object recognition models are designed to classify the entire image without explicitly isolating the target object from its background.

Notably, Borji (2021) showed that removing the background via human-generated bounding boxes improved DCNN performance on ObjectNet by 20–30% compared to full-image presentations, also suggesting that context strongly influences recognition. Moreover, the challenge arises when objects appear in atypical real-world settings—such as incongruent backgrounds, unusual orientations, or combinations of these factors—which deviate from the statistical regularities learned during training.

Importantly, the contextual complexity of ObjectNet is central to the aims of the present study rather than a drawback. In ObjectNet, objects often appear in unusual real-world settings that diverge from the statistical patterns typically found in the standard IID benchmark test set. Given that DNNs are known to rely on shortcut associations learned from these regularities in standard training data such as ImageNet, ObjectNet limits the extent to which models can succeed by exploiting trivial correlations unrelated to object identity. This property is particularly relevant in light of recent claims that contemporary models match or exceed human-level performance, as ObjectNet provides a more realistic benchmark for assessing whether such claims hold under varied, cluttered, and incongruent real-world conditions. In such OOD scenarios, full-image presentations can compound context-dependent biases in both humans and machines, although the effects and underlying mechanisms may differ between the two systems.

An often-overlooked factor in human–machine comparisons is individual variability in human cognitive traits. Evidence shows substantial differences in personality and cognitive abilities (Richler et al., 2019), meaning that assuming uniform object recognition ability across individuals—and comparing machines to aggregated human averages—can yield misleading conclusions. Averaging data can obscure variability and unique individual patterns, concealing critical behavioural differences. As Estes (1956) highlighted, aggregated data can obscure individual-level differences, suggesting group-level patterns or trends that may not actually exist at the individual level. Comparing human and machine categorisation behaviour patterns without accounting for individual variability can lead to misinterpretation.

### Current Study

This study compares object recognition in humans and a range of machine vision models, from traditional DCNNs to state-of-the-art (SOTA) multimodal systems, under balanced experimental conditions. A key contribution of this work is the inclusion of models spanning multiple generations of architecture—from DCNNs to vision transformers and, importantly, the recent multimodal CoCa model (Yu et al., 2022)—to assess how far current systems align with human categorisation behaviour beyond overall accuracy. In addition, the study provides a novel analysis by examining individual differences in human categorisation performance, identifying subgroups and comparing their patterns with those of the models, rather than relying solely on group-level accuracy.

Following Firestone’s (2020) recommendations, we imposed similar constraints on both systems and evaluated not only accuracy but also error patterns, response profiles, and the alignment of categorisation behaviour with that of humans, accounting for individual differences.

Experiment 1 uses N-way categorisation for humans and models on a subset of ObjectNet to test whether models can match or exceed human accuracy under time constraints.

Experiment 2 compares free-naming in humans and models under two conditions—isolated objects (bounding boxes) and full-scene images—to assess the influence of background context. We expect background removal to improve performance, particularly for models, while human performance will vary with context congruence.

Experiment 3 examines individual differences in brief-presentation recognition, identifies human subgroups and compares their patterns with model behaviour.

Together, these experiments provide a multidimensional comparison of object recognition across humans and machine models, at both group and individual levels.

## 2. Experiment 1

We assessed human performance under two masked-stimulus presentation times—50 ms and 200 ms. The 200 ms duration was selected because processing in the human visual system is likely still predominantly feed-forward at this timescale (DiCarlo et al., 2012; Geirhos et al., 2018; Serre et al., 2007). This is relevant given that the DCNNs used for comparison are strictly feed-forward, whereas, at longer durations, human perception makes use of both feed-forward and feedback connections (Cox & Dean, 2014). Hence, rather than providing an upper bound on human performance, our 200 ms condition provides a comparison where both machine and (likely) human depend on feed-forward processes. Masking further minimises the contribution of feedback after stimulus offset (Serre et al., 2007). The 50 ms condition was chosen as it approximates the limen of conscious perception in humans (see, e.g., Marcel, 1983). To ensure a fair comparison, the DNNs in Experiment 1 were limited to performing the same 10-alternative forced-choice task as human participants, with model outputs restricted to the same ten object categories used in the human multiple-choice experiment.

### 2.1. Experiment 1: Method (Humans)

#### 2.1.1. Stimuli

Our stimuli were a subset of the large ObjectNet image set (https://objectnet.dev, accessed on 10 December 2025), which comprises pictures of everyday items such as a mug (Barbu et al., 2019). We selected 10 categories that spanned the range of difficulties for the DCNNs under test (see Section 2.2 Experiment 1: *Method (machines)*, based on the models’ performance in classifying ObjectNet categories using TensorFlow 2.4.1 (Abadi et al., 2016). The difficulty levels for each category were determined by ranking the 113 ImageNet-overlapping categories according to the accuracy achieved by each DCNN, from highest to lowest. We then identified categories that consistently appeared in the worst-performing, mid-performing, and best-performing groups across the DCNNs. Based on these trends, we selected three difficult categories (Plate, Vase, Basket), four moderate categories (TV, Doormat, Helmet, Mug), and three easy categories (Drill, Plunger, Safety pin). Note that human performance was not taken into account when assessing the difficulty of these categories.

Each category in the ObjectNet image set contains more than 100 images. We excluded images in which the labelled object was partially out of frame, occluded, blurred, or in which another object occupied the central position. After excluding poor-quality images, we arbitrarily selected 60 images per category (600 images in total), as using a larger set of images would likely have led to participant fatigue.

#### 2.1.2. Participants

A total of 25 adult participants from the School of Psychology, University of Plymouth, UK, participated voluntarily and received participant points for their participation. All participants had normal or corrected visual acuity.

#### 2.1.3. Apparatus

The experiment was run using OpenSesame 3.3.11b1 (Mathôt et al., 2012) on a Lenovo ThinkPad laptop with a 15.6-inch screen. All participants were tested individually in the presence of one of the authors (GK).

#### 2.1.4. Procedure

Participants were assumed to be pre-experimentally familiar with the categories; hence, the experiment consisted entirely of a test phase.

Each trial began with the presentation of a central fixation point (500 ms). The image was then presented to participants for randomised presentation times (200 ms or 50 ms), followed by a pink mask (for 200 ms). Participants then made a self-paced response. The name of each object category and its corresponding number were displayed on the screen each time, and participants responded by pressing a number key (1–9 or 0) on the keyboard corresponding to the category they believed was correct.

Each of the 600 images was presented once at each presentation time (200 ms and 50 ms), in random order, for a total of 1200 trials across eight blocks. Participants were able to rest between blocks.

#### 2.1.5. Assessing Potential Carryover Effects

A within-subjects design was used in which participants completed both presentation-time conditions (50 ms and 200 ms). Each of the 600 images was shown once at each duration, resulting in 1200 trials per participant, distributed across eight blocks (150 trials per block). Each image was presented twice—once at 50 ms and once at 200 ms—so the assignment of presentation durations to object categories was counterbalanced across blocks to minimise potential systematic bias. In Blocks 1–4, five categories were shown at 50 ms and the other five at 200 ms; in Blocks 5–8, this mapping was reversed. However, despite this counterbalancing, the repeated presentation of images still introduces the possibility of carryover effects in the human data, such as repetition priming or memory-based responding (Berry et al., 2017).

To assess whether repeated exposure influenced performance, we conducted a follow-up analysis. For each participant and condition (50 ms and 200 ms), mean classification accuracy was computed separately for the first and second halves of the experiment, corresponding to the first and second image presentations. A Bayesian paired-samples *t*-test was then used to compare performance between the two halves within each presentation time condition. The results provided no evidence of carryover effects. For the 50 ms condition, the Bayes Factor was BF = 0.23, indicating moderate evidence in favour of the null hypothesis. For the 200 ms condition, the Bayes Factor was BF = 0.46, indicating anecdotal evidence for no effect. These findings show performance was not systematically improved by repetition, supporting the validity of comparing human and DNN performance in this experiment.

### 2.2. Experiment 1: Method (Machines)

All DNNs were fine-tuned using a subset of the ImageNet training set—except CoCa (Yu et al., 2022), tested with task-specific zero-shot classification (see below)—and then evaluated on the same ObjectNet images presented to human participants. Full details on model selection are in Appendix A.

Before comparing the DNNs with our human participants, the DNNs were “fine-tuned” for better performance on the ten categories used in our experiment. Fine-tuning involves performing “surgery” on pre-trained DCNNs by cutting off the classification head and placing a new classification head on top of the architecture of neural networks. Instead of end-to-end fine-tuning, we fine-tuned only the classifier head of the models. Except for the new classification head, all layers were frozen; therefore, the weights of these layers cannot be updated, allowing only the new classification head to learn patterns from the convolutional layers (Rosebrock, 2017). All the DCNN models in this thesis have been trained on the 1000 classes of the ImageNet image set. In these models, the classification head corresponds to the output of the 1000-way ImageNet classification task, which produces a distribution over the 1000 object class labels; likewise, the classification head of ViT models was fine-tuned on these 1000 classes. In this case, the classification head of these models, corresponding to the ImageNet thousand-way object classification task, was replaced with an eleven-category output layer, with one node per category in this experiment. The reason for reducing the number of output neurons from 1000 to 11, to match the ten entry-level classes instead of 10, is that the ‘Helmet’ category was mapped into two categories in the ImageNet dataset: “Crash helmet” and “Football helmet”.

Following these modifications, we further trained the models on the ImageNet training set for only these eleven categories. We used 90% of the images in each category for training and reserved the remaining 10% for validation. The remaining training steps followed standard procedures for fine-tuning the classifier head of supervised pre-trained DNNs on ImageNet: we used Adam (Kingma & Ba, 2014) as an optimiser, a batch size of 32, and a learning rate of 0.0001 for 25 epochs. Fine-tuning was done using Pytorch 2.0.1 (Paszke et al., 2019).

All DNNs in this study were fine-tuned, except for the CoCa model, which was not included in this process due to practical reasons. The CoCa model was pre-trained using a self-supervised learning approach. Unlike traditional DNNs with a classification head, CoCa lacks this feature; therefore, we used a zero-shot classification procedure to evaluate the CoCa model. In zero-shot classification, the model assigns predefined class labels to images without any task-specific training by comparing image representations with textual descriptions of each category. Since CoCa learns a joint image–text representation during pretraining, it can recognise and categorise images based solely on natural language prompts, even for categories it has not encountered before. This approach enabled CoCa to classify images into the same 11 categories used by the other models, analogous to how a classifier head generates responses from a distribution over class labels. We utilised the zero-shot image classification pipeline from the OpenCLIP GitHub repository (Ilharco et al., 2021), using the version available as of June 2024, which provides a PyTorch implementation of unsupervised representation learning methods, including pre-trained representation weights, training, and evaluation pipelines. We applied this evaluation pipeline to test CoCa on the same ObjectNet images used with other DNNs and human participants, using labels for 11 ImageNet object classes and the exact prompt texts provided by the repository.

After fine-tuning DNNs on a subset of the ImageNet training set, except for the CoCa model, the accuracy of all DNNs was assessed against the same set of ObjectNet images presented to human participants.

### 2.3. Experiment 1: Results

Statistical analysis was performed in the R environment (R Core Team, 2023) using the BayesFactor package (Morey & Rouder, 2022).

#### 2.3.1. Humans

**Figure 2:**
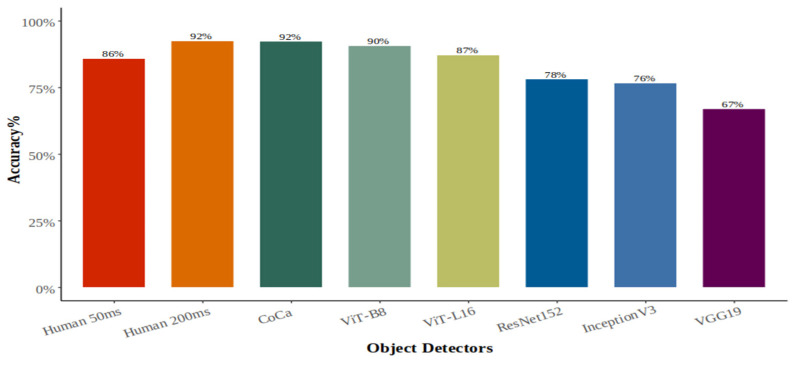
Average accuracy % of responses for DNNs and 25 participants at each stimulus presentation time.

**Figure 3:**
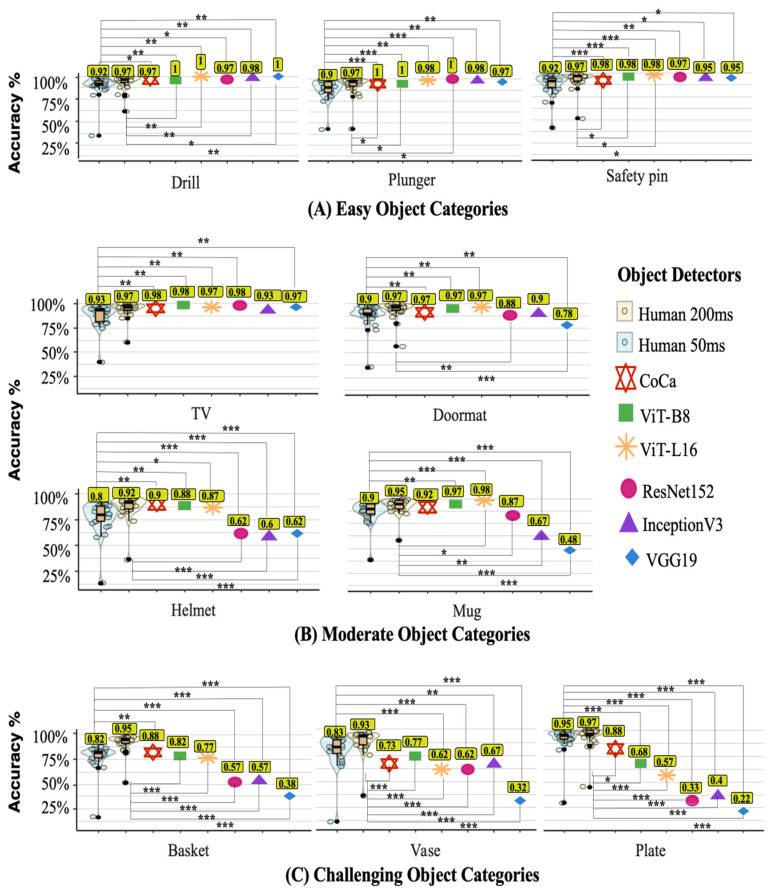
Accuracy of humans and the artificial vision systems across object categories of varying difficulty: easy (Panel (**A**)), moderate (Panel (**B**)), and challenging (Panel (**C**)) object categories. Human 50 ms and Human 200 ms denote human accuracy at different stimulus presentation times; CoCa, ViT-B8, ViT-L16, ResNet152, InceptionV3, and VGG19 denote artificial vision systems. The box plot (filled in orange) showcases the median performance and interquartile range of human data, with solid black circles indicating potential outliers identified using the interquartile range criterion. Significance lines indicate comparisons between human and model accuracies: comparisons to Human 50 ms are shown above the boxplots, and comparisons to Human 200 ms are shown below. Different signifiers indicate varying levels of Bayesian evidence for differences, with * indicating BF > 3, ** indicating BF > 10, and *** indicating BF > 100.

As shown in Figure 2, although accuracy decreased slightly at the 50 ms presentation time (86%) relative to the 200 ms condition (92%), humans performed at a high level of accuracy in both conditions of this task. There is strong Bayesian evidence for the difference between these two conditions, BF = 1.17 × 10^26^. There is also strong evidence for a main effect of object category (BF = 8.8 × 10^23^), and for an interaction between presentation time and object category (BF = 1.82 × 10^6^). These latter two effects are illustrated in Figure 3.

As shown in Figure 2, human performance at 200 ms exceeded that of all DCNNs and ViTs except CoCa. Bayesian evidence strongly supported these differences for the DCNNs (BFs > 1000) and for ViT-L16 (BF = 4.58), while there was some evidence for the absence of a difference with ViT-B8 (BF = 0.30) and CoCa (BF = 0.20). At 50 ms, humans outperformed all DCNNs, with Bayes factors indicating moderate to extreme evidence (BFs > 3). In contrast, CoCa and both ViT models outperformed humans numerically at 50 ms. However, the evidence for these differences was weak for CoCa (BF = 3), inconclusive for ViT-B8 (BF = 1), and provided some evidence for the absence of a difference for ViT-L16 (BF = 0.24). Across conditions, CoCa achieved the highest accuracy, outperforming humans at 50 ms, matching human performance at 200 ms, and surpassing all other DNNs.

#### 2.3.2. Human–Machine Comparison (Category-Level Accuracy)

As depicted in Figure 3A, for the drill, plunger, and safety pin categories that the DCNNs found easy, all DNNs numerically surpassed humans at 50 ms and either matched or surpassed humans at 200 ms. Formal comparisons between the DNNs and human performance provided Bayesian evidence that supports this trend at 50 ms (BF > 3; see Figure 3A for significance levels of Bayesian evidence for each easy category). Notably, only ViT-B8 outperformed humans numerically and formally across all easy categories at 50 ms and 200 ms.

In Figure 3B, the Doormat, Helmet, Mug, and TV categories are moderately challenging for DCNNs. Overall category accuracies and the Bayesian evidence for differences between human and model accuracies yield mixed results. Vision transformer-based models outperformed humans at both 50 ms and 200 ms. When comparing these highly accurate ViT models with human performance, the Bayesian evidence supports this pattern for humans at 50 ms (3 < BF < 100) but for humans at 200 ms only with the ViT-L16 in the mug category, BF = 8.69.

Figure 3C illustrates the performance of humans and machine learning models for the basket, plate, and vase categories—objects that were particularly challenging for DCNNs. Across these categories, humans significantly surpass these models, a conclusion strongly supported by overwhelming Bayesian evidence. Only CoCa formally and numerically performed better than humans in the basket category, making it the only Bayes Factor result favouring DNNs over humans in the most challenging categories at both 50 ms and 200 ms.

#### 2.3.3. Trial-by-Trial Agreement Analysis

To assess whether models and humans made similar classification decisions beyond accuracy alone, we conducted a trial-by-trial inter-rater agreement analysis using Cohen’s Kappa coefficient (Cohen, 1968) to compare machines with machines, humans with humans, and machines with humans. This measure accounts for chance agreement, with κ values ranging from −1 (perfect disagreement) to 1 (perfect agreement). Following Landis and Koch’s (1977) interpretation, values ≤ 0 indicate no agreement, 0.01–0.21 slight, 0.21–0.40 fair, 0.41–0.60 moderate, 0.61–0.80 substantial, and 0.81–1.00 almost perfect agreement. Unweighted κ was calculated using the R “irr” package (Gamer et al., 2010).

**Figure 4:**
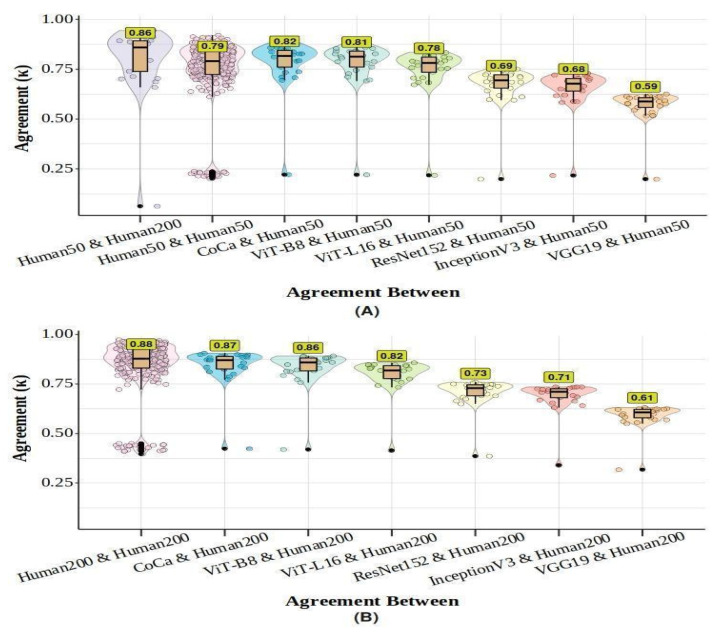
Distribution of overall agreement (unweighted Kappa-values) for DNNs and human participants across 50 ms and 200 ms presentation times. Panel (**A**) displays the overall agreement distribution for each participant with each other and with each DNN in the 50 ms condition. Additionally, the comparison of each individual’s performance at 50 ms with their performance at 200 ms is included. Panel (**B**) illustrates the overall agreement distribution of each DNN with each participant and each participant with each other in the 200 ms condition. The median Kappa values for each comparison group are emphasised in yellow boxes. Distributional information is presented as a box plot, a violin plot, and individual data points. The box plot showcases the median performance and interquartile range, with solid black circles indicating potential outliers identified using the interquartile range criterion.

Overall, agreement was high across classifiers. Table 1 shows that ViTs and CoCa models had the highest level of agreement with each other (0.87 < κ < 0.88), while VGG19 had the lowest agreement with both DNNs and humans (0.59 < κ < 0.68). Figure 4 illustrates the level of agreement between humans and the agreement between DNNs and humans. Human–human agreement was substantial at 50 ms (κ = 0.79) and almost perfect at 200 ms (κ = 0.88). CoCa demonstrated the highest human–model agreement, suggesting it most closely replicated human response patterns, followed by ViTs, which were more human-like than DCNNs. VGG19 exhibited the least similarity to humans. Overall, the latest SOTA models, particularly CoCa, displayed more human-like classification behaviour than DCNNs.

### 2.4. Experiment 1: Discussion

Although humans and DNNs achieved comparable overall accuracy in Experiment 1, machine performance lagged substantially behind humans for certain everyday, basic-level categories—most notably basket, vase, and plate. Prior research suggests that such differences may arise from humans, but not DNNs, relying on three-dimensional, object-centred representations (Alcorn et al., 2019; Borji & Itti, 2014; Cooper et al., 2021). For example, plate recognition poses challenges for models due to the high variability of 2D appearance under rotation, in which local texture or colour cues may be less effective.

When comparing architectures, advanced vision models such as CoCa exhibited the closest alignment with humans in terms of both overall accuracy and response patterns. However, while higher accuracy can lead to greater agreement with human responses, this does not necessarily indicate that error patterns and decision strategies are similar—especially when evaluating misclassifications. Therefore, any agreement should be interpreted with caution, as discussed further in the Section 5 along with other findings from this experiment and their broader implications.

## 3. Experiment 2

This experiment compared human and machine performance under two conditions: isolated objects and full-image presentations. Machine performance was evaluated using DNNs trained on the full 1000 ImageNet categories, retaining their original classification outputs rather than fine-tuning them for the task-specific categories used in Experiment 1. While fine-tuning can improve machine accuracy, it introduces additional training biases and grants an advantage to models over humans. By using pre-trained models without task-specific tuning, we aimed for a more equitable comparison with human recognition in a free-naming paradigm. We could not implement a true free-naming procedure for the models because their architectures are constrained to a fixed 1000-way classification output. As a result, models selected from the 1000 ImageNet categories, whereas human participants were not limited in their naming responses, leading to greater variability in individual responses.

Human participants named objects after a brief presentation (50 ms) followed by a pink-noise mask, ensuring responses reflected rapid feed-forward processing. This protocol followed the same rationale as Experiment 1. To establish an upper bound on human performance, a separate group completed the task without time constraints, thereby enabling both feed-forward and recurrent visual processes. Performance in this unrestricted condition served as a benchmark for evaluating the 50 ms condition and model outputs, in which both machines and (likely) humans depend on feed-forward processes.

### 3.1. Experiment 2: Method (Humans)

#### 3.1.1. Stimuli

We aimed to address methodological issues related to the object categories and images used in Experiment 1, including instances in which specific objects were misclassified in ObjectNet. Improvements to the stimuli and experimental design were implemented through a re-naming study involving eight volunteers, ensuring a more robust and valid comparison of human and machine performance across conditions. Based on these findings, we selected nine categories—arbitrarily selecting 30 images per category, all of which achieved 100% naming agreement among all eight participants for each category—classified into three difficulty levels: Difficult (Plate, Vase, Basket), Moderate (TV, Helmet, Mug), and Easy (Banana, Match, Plunger).

#### 3.1.2. Participants

A total of 25 participants were recruited in each of the two experimental conditions (50 ms presentation time and self-paced). All participants were from the School of Psychology, University of Plymouth, UK, and participated voluntarily, receiving participant points for their participation. All participants had normal or corrected visual acuity.

#### 3.1.3. Apparatus and Procedure

The experimental procedure and apparatus were similar to those in Experiment 1, with a few key differences. Instead of employing a forced-choice categorisation task with presentation times of 50 ms and 200 ms as in Experiment 1, this experiment utilised a free-naming paradigm with two presentation-time conditions: the 50 ms condition and the self-paced viewing condition (referred to as the “self-paced group” throughout the thesis). In the 50 ms condition, each image was displayed for 50 ms, followed by a 200 ms pink mask. In the self-paced group, participants viewed each image at their own pace, pressing any key when ready to proceed to the response form, also followed by a 200 ms pink mask. After the mask, participants in both conditions were asked to “type the name of the object with ONLY ONE WORD that stands out” to them via the keyboard. No feedback was provided.

A total of 270 images, equally divided between isolated-object and full-image conditions, were presented once in random order. Both the 50 ms and self-paced groups completed 270 trials in total, spread over six blocks. Participants were allowed to take breaks between blocks.

### 3.2. Experiment 2: Method (Machines)

The accuracy of all DNNs in Experiment 2 was evaluated using PyTorch 2.0.1 (Paszke et al., 2019) on the same set of ObjectNet images used in the human experiments across both isolated and full-image conditions. The models’ performance was assessed using Top-1 accuracy, which is the proportion of trials in which the model’s top prediction—selected from 1000 possible categories—matches the correct object label. CoCa was evaluated using labels for 1000 ImageNet object classes and exact prompt texts provided by the OpenCLIP GitHub repository. This approach enabled CoCa to produce classification responses from among these 1000 categories, similar to how the classifier head of other models generates responses based on a distribution over 1000 object class labels in classification tasks.

### 3.3. Experiment 2: Results

We applied the same statistical analysis and methods as in Experiment 1. For further details on Accuracy coding and response correction in the free-naming task, see Appendix B.1.

#### 3.3.1. Humans

**Figure 5:**
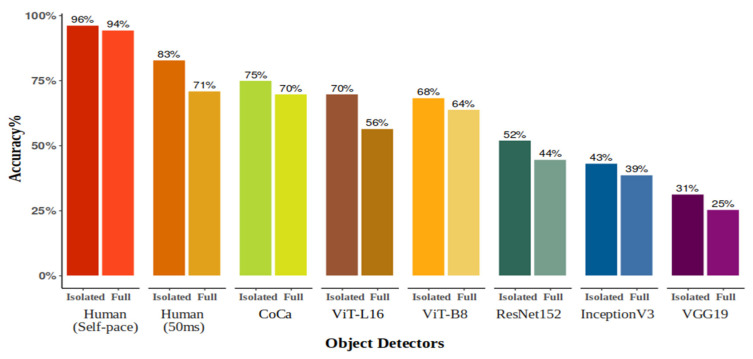
Average accuracy % of responses for DNNs and 25 participants at both 50 ms presentation time and self-paced viewing for each experimental condition—images cropped according to bounding box coordinates (Isolated) and images with entire scenes (Full).

As shown in Figure 5, humans performed better without time limits (self-paced) than at 50 ms. Self-paced accuracy was near-perfect for both isolated objects (96%) and full images (94%). At 50 ms, accuracy dropped by 12% for full images (71%) compared with isolated objects (83%), although humans performed at reasonably high accuracy in both conditions. Two-way mixed Bayesian ANOVAs for each image condition showed extreme evidence for a main effect of presentation time (isolated: BF = 3.14 × 10^22^; full: BF = 2.63 × 10^31^) and significant interactions between object category and presentation time (isolated: BF = 6.03 × 10^8^; full: BF = 5.25 × 10^24^).

#### 3.3.2. Human–Machine Comparison (Overall Accuracy)

As illustrated in Figure 5, CoCa achieved the highest DNN accuracy for both isolated objects (75%) and full images (70%), with ViTs following closely behind. Among CNNs, ResNet-152 achieved the best performance, whereas VGG-19 had the lowest. Humans outperformed all models in both self-paced and 50 ms conditions, even surpassing CoCa in the 50 ms condition. Bayesian two-sample *t*-tests provided strong evidence (BFs > 3) that humans outperformed all models in the 50 ms isolated condition, and all except CoCa (BF = 0.25) in the full-image condition. In the self-paced condition, humans showed extreme evidence of superior accuracy (BFs > 1 × 10^12^) across both image types.

#### 3.3.3. Human–Machine Comparison (Category-Level Accuracy)

Category-level comparisons between humans and models for easy, moderate, and challenging object categories are shown in Supplementary Figures S1–S3, with isolated and full-image conditions presented separately. Numerically, some models—mainly vision transformer-based models (ViTs and CoCa)—surpassed humans in categories classified as easy and moderate, while humans substantially outperformed models in harder categories.

Overall, for machine-easy categories, humans in the self-paced condition outperformed all DNNs across both image conditions, with only minor exceptions where transformer-based models (CoCa and ViTs) slightly exceeded human accuracy for isolated banana objects. In the 50 ms condition, ViTs often outperformed humans, except for isolated match objects, and CoCa showed a similar pattern, with exceptions for isolated match and plunger objects. Compared with DCNNs, humans generally performed better, except in full-image cases, in which ResNet152 achieved higher accuracy. A detailed breakdown of the Bayesian evidence and performance patterns is provided in Supplementary Figure S1.

For the moderate categories, humans in the self-paced condition outperformed all DNNs across both image conditions, with only minor exceptions: CoCa marginally exceeded human performance for isolated banana objects, whereas ViT-B8 outperformed humans in full-helmet images. At 50 ms, humans outperformed models in the mug and TV categories across both image conditions, except for CoCa in isolated mug objects. In contrast, transformer-based models surpassed human accuracy in the helmet category for both image conditions. This category was notably the most challenging for humans at 50 ms. Among DCNNs, ResNet152 exceeded human performance only for full-helmet images. Detailed Bayesian evidence and performance patterns are shown in Supplementary Figure S2.

For machine-challenging categories, humans in the self-paced condition outperformed all DNNs across both image conditions. At 50 ms, humans generally maintained this advantage, with exceptions for CoCa in isolated plate images and ViT models in full vase images. Vase accuracy at 50 ms was the lowest for humans after the helmet category. When humans outperformed the models, the Bayesian evidence was extreme; conversely, when models outperformed humans, the evidence was weak or inconclusive. Full results are shown in Supplementary Figure S3.

#### 3.3.4. Trial-by-Trial Agreement Analysis

We applied the same trial-by-trial inter-rater agreement analysis, using the same methods as in Experiment 1.

**Figure 6:**
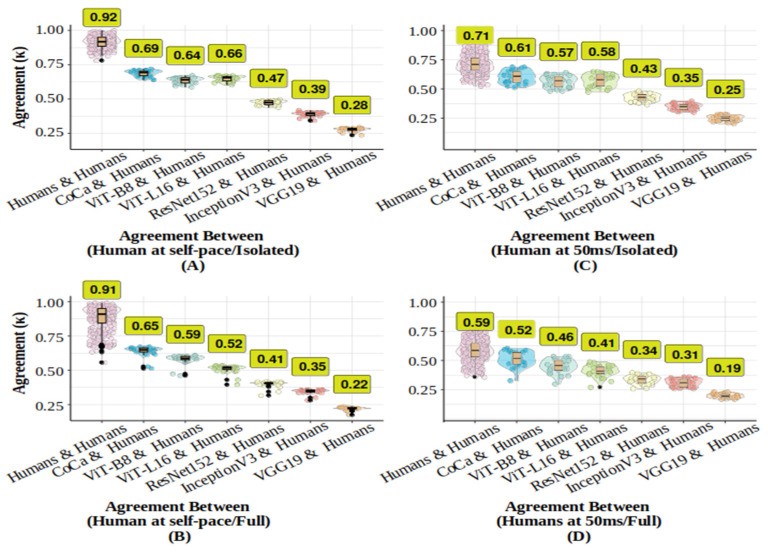
The four panels display the distribution of overall agreement (unweighted Kappa Values) for DNNs and human participants under 50 ms and self-paced viewing, across isolated-object and full-image conditions. (**A**) The overall agreement distribution for each participant with each other and with each DNN in the isolated objects condition for the self-paced condition. (**B**) The overall agreement distribution for each participant with each other and with each DNN in the isolated objects condition for the 50 ms condition. (**C**) The overall agreement distribution for each participant with each other and with each DNN in the full-image condition for the self-paced condition. (**D**) The overall agreement distribution for each participant with each other and with each DNN in the full-image condition for the 50 ms condition. The median Kappa values for each comparison group are emphasised in yellow boxes. Distributional information is presented as a box plot, a violin plot, and individual data points. The box plot showcases the median performance and interquartile range, with solid black circles indicating potential outliers identified using the interquartile range criterion.

Across both image conditions, the overall agreement of response patterns was highest among humans in the self-paced condition, with near-perfect alignment, and declined under the 50 ms presentation condition, particularly for full images. Across models, agreement with humans was generally higher in isolated-object conditions than in full images, with CoCa showing the strongest alignment across both presentation times, followed by ViTs. DCNNs exhibited the lowest agreement with human responses, with ResNet152 performing best, whereas VGG19 showed the weakest alignment. Agreement among models followed a similar pattern: transformer-based models showed the highest agreement with one another compared to other machine learning models. For detailed values and pairwise comparisons, see Table 2 and Figure 6.

### 3.4. Experiment 2: Discussion

This study found that both humans and DNNs achieved higher accuracy when recognising isolated objects than when recognising objects presented in cluttered, context-rich scenes, indicating that background context influences object recognition in both systems. One notable pattern in the human data was the marked decline in accuracy for certain categories, particularly helmet, under the 50 ms full-image condition. Misclassification patterns, partly due to increased response variability in the free-naming task, showed that helmets were frequently labelled as balls, mugs, bowls, or plates. This decrease in accuracy can be attributed to the fact that, during very brief exposure, humans tend to depend on rapid, low-level visual features such as overall shape and size (Serre et al., 2007). Since helmets share common visual characteristics with objects like balls and bowls—rounded shapes, smooth surfaces, and similar proportions—they are more vulnerable to misclassification, especially under rapid viewing conditions. Background clutter and scene context may further contribute to these errors: when a helmet appeared in an unexpected or incongruent setting, rapid contextual associations may have biased observers toward more context-typical objects—for instance, a helmet appearing in a kitchen-like setting being classified as a mug or plate (Murphy et al., 2006). Together, the combination of ambiguous shape, atypical contexts, and minimal processing time likely drove the reduced human accuracy.

Among the models, advanced vision systems such as CoCa exhibited the greatest similarity to human response patterns. Nonetheless, the robustness of human visual processing in complex, real-world-like scenarios underscores a key advantage over machine vision, particularly when models are evaluated without fine-tuning for the task. The findings of Experiment 2, and their broader implications for human–machine comparisons in naturalistic object recognition, are elaborated in the Section 5.

## 4. Experiment 3

Experiment 3 compared human and machine performance across nine object categories, focusing on both aggregate quantitative accuracy and individual-level ordinal performance patterns, including variability among humans. Before analysing individual differences, we assessed the reliability of human performance measures using a test–retest design to evaluate intra- and inter-rater consistency across two sessions, ensuring that the measures used to evaluate whether human performance is reliable at the individual level, because the systematic study of individual differences requires first showing that such variability reflects stable traits or abilities, rather than just measurement error (Gauthier, 2018; Hedge et al., 2018). To minimise fatigue and maintain data quality, both sessions used a single presentation duration of 50 ms followed by a 200 ms pink-noise mask—chosen for its proximity to the threshold of conscious perception and its reliance on feedforward visual processing while limiting recurrent feedback. Humans and models were both tested using an N-way categorisation procedure similar to Experiment 1. This involved a multiple-choice process across nine object categories for the humans. In contrast, for the models, we fine-tuned the classification head of DNNs to ensure their outputs aligned with the nine category options.

### 4.1. Experiment 3: Method (Humans)

#### 4.1.1. Stimuli

In this experiment, we used the same category and stimulus set as in Experiment 2, comprising nine object categories divided into three levels of difficulty: Difficult (Plate, Vase, Basket), Moderate (TV, Helmet, Mug), and Easy (Banana, Match, Plunger), for a total of 270 images—each image was presented only in its full-image form.

#### 4.1.2. Participants

A total of 398 adult participants from the School of Psychology, University of Plymouth, UK, voluntarily participated online through the SONA (Sona Systems, n.d.) recruitment system and received participant points for doing so. All participants had normal or corrected visual acuity.

#### 4.1.3. Apparatus

The experiment was programmed in OpenSesame 3.3.11b1 (Mathôt et al., 2012) to be run in a web browser and then deployed through the JATOS server (Lange et al., 2015). JATOS allows participants to join a study smoothly via a single-user link, ensures no duplicate submissions, and securely stores all data. The experiment automatically ran in a web browser after participants accessed the study through the single-user link. Before the participants clicked the experiment link, they were asked to complete the study in a quiet room and avoid distractions. Participants completed the experiment on their personal computers. The experiment lasted, on average, approximately 18 min. While most participants completed the task within 30 min, four participants showed substantially longer total durations, likely due to pauses or leaving the browser open during the session.

#### 4.1.4. Procedure

The procedure closely followed the protocol from Experiment 1: The presentation time for all stimuli was fixed at 50 ms instead of 50 ms or 200 ms, and participants completed the task twice to calculate test–retest reliability. Human trials are structured into a practice phase, testing, and retesting stages. The practice phase comprised five trials; each stimulus was presented for 50 ms, as in Experiment 1. This phase ensured that participants understood the rapid presentation format and needed to maintain focus to avoid missing stimuli. Participants were informed that the main experiment consisted of 12 blocks, each with 45 stimuli; after completing six blocks, there would be a 5 min mandatory break, marking the midpoint of the experiment. Participants were not told that the same images from the test session would be presented again during the retest session.

During the test session, all 270 images were presented once at 50 ms, followed by a 200 ms pink-noise mask. Stimuli were presented in random order, distributed evenly across six blocks, resulting in a total of 270 trials. In the retest session, the same 270 images were presented again in the same manner as in the test session. Overall, participants completed 540 trials across 12 blocks; they could rest between blocks.

After completing the test session, participants were given a mandatory 5 min break before proceeding to the retest session. To ensure that participants remained within this time frame—considering they might leave the computer and forget about the experiment—they were required to engage in an anagram game that tested their ability to rearrange jumbled letters. For example, the letters “GDERFI” can be rearranged to form the word “FRIDGE”. Participants were asked to solve as many anagrams as possible within the time limit.

A countdown timer set to five minutes was clearly displayed at the top centre of each slide, showing minutes and seconds. As participants progressed to the next anagram slide, the countdown allowed them to track the remaining time. Participants progressed through up to 200 anagram slides, ensuring that the activity would extend beyond the 5 min break time even if they skipped all questions. After the countdown reached zero, participants proceeded to the information page for the second part of the task (retest session).

During both the test and retest sessions, all participants completed each block in the same order. Additionally, an identical set of images was displayed within each block for all participants to maintain consistency and avoid potential participant-order interactions that could influence the results.

### 4.2. Experiment 3: Method (Machines)

Experiment 3 follows the same methodology as Experiment 1, fine-tuning the classification heads of ImageNet pre-trained DNNs and evaluating the accuracy of the same object-recognition algorithms. To match the human multiple-choice procedure, the classification heads of the DNNs were fine-tuned on a subset of the ImageNet training set corresponding to our nine ObjectNet categories, resulting in nine category options, as in Experiment 1. The same nine object categories at three difficulty levels, a total of 270 images—each presented only in full-image form—were used from Experiment 2.

### 4.3. Experiment 3: Results

We applied the same Bayesian statistical analyses as in Experiments 1 and 2. For human–machine comparisons, only participants’ initial test session data were used, as the retest session was intended to assess reliability. Furthermore, retest performance could be influenced by task familiarity or reduced motivation after the initial session, potentially confounding the comparison.

#### 4.3.1. Humans

The overall mean percentage accuracy for the test session was 86%, indicating that humans performed the task well. The average human accuracy across all object categories exceeds 75%, with the only exception being the helmet category in the test session (74%).

A Bayesian one-way within-subjects ANOVA was conducted for the test session, and the results provided extreme Bayesian evidence for the effect of object category on human accuracy, with a Bayes Factor of 7.9 × 10^456^.

#### 4.3.2. Human–Machine Comparison (Overall Accuracy)

**Figure 7:**
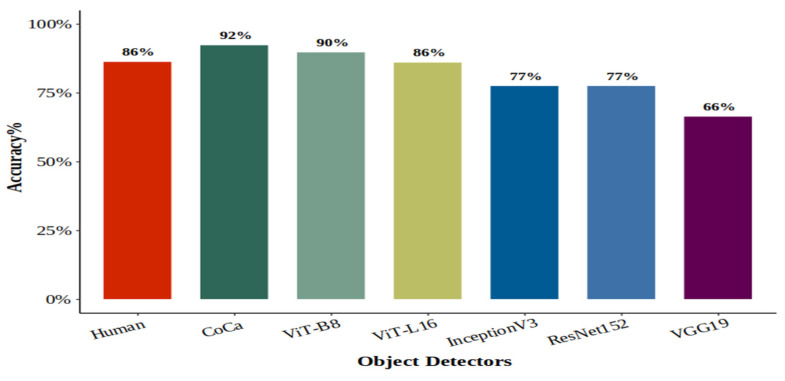
Average accuracy % of responses for DNNs and 398 participants in the test session at each stimulus presentation time.

In terms of overall accuracy, the top-performing vision models, CoCa and ViT-B8, numerically and formally outperformed both humans and all the DNNs, each with a BF > 1000. In comparison, human performance was higher than that of ViT-L16 and all the DCNNs—InceptionV3, ResNet152 and VGG19 (see Figure 7). Bayesian *t*-test analysis provided extreme evidence favouring human performance over DCNN models (BF > 1000). However, the Bayes Factor for the difference between ViT-L16 and humans indicated moderate evidence for the null hypothesis (BF = 0.06), suggesting that ViT-L16 performs similarly to humans and provides no strong evidence of superiority.

#### 4.3.3. Human–Machine Comparison (Category-Level Accuracy)

Category-level comparisons between humans and models for easy, moderate, and challenging object categories are shown in Supplementary Figures S4, S5 and S6, respectively.

For the banana, match, and plunger categories that the models find easy, only ViT-B8 numerically and statistically exceeded human performance, with each Bayes Factor greater than 100. In a few cases, human performance exceeded that of machine learning models, both statistically and numerically (see Supplementary Figure S4 for the strength of Bayesian evidence associated with each easy-level category).

The TV, helmet, and mug are considered moderately difficult categories for the models. Vision transformer-based models, such as CoCa and ViTs, demonstrated superior performance relative to humans across all moderate categories, supported by extreme Bayesian evidence (BFs > 100). In contrast, convolutional models—InceptionV3, ResNet152, and VGG19—exceeded human accuracy only in the TV category (BFs > 10; see Supplementary Figure S5 for details on the Bayesian evidence and performance patterns).

For machine-challenging categories, convolutional models struggled most with the plate, vase, and basket categories, whereas humans outperformed these models numerically and significantly across all challenging categories. However, vision transformer-based models, notably CoCa, demonstrated performance comparable to or better than that of humans in these challenging categories, particularly in the basket and vase categories. Full results are shown in Supplementary Figure S6.

#### 4.3.4. Test–Retest Reliability and Trial-by-Trial Agreement Analysis

Kappa values were computed and interpreted according to Landis and Koch’s (1977) guidelines, as outlined in Experiment 1, with 95% confidence intervals computed using the “psych” package (Revelle, 2024).

**Figure 8:**
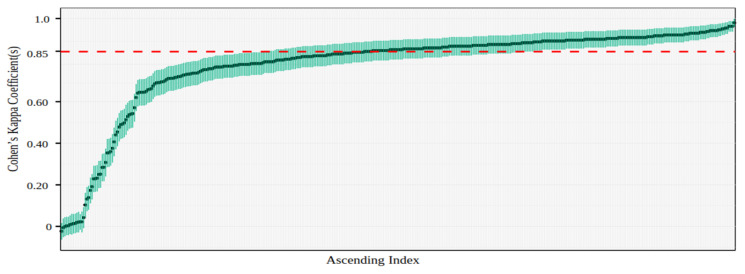
The dotted line is the median Kappa value of test–retest reliability. Each dot with a 95% confidence interval bar corresponds to a Kappa value representing how much a participant agreed with themselves. There is an almost tenfold difference between the largest and the smallest Cohen’s Kappa Coefficients.

Participants performed well in both sessions (test: *M* = 86.2%, *SD* = 13.5; retest: *M* = 85.2%, *SD* = 18.6), with no evidence for a difference between sessions (BF < 3). Participants showed high intra-rater reliability across sessions (median κ = 0.85), with 287 of 398 achieving substantial or near-perfect agreement (see Figure 8). Overall, individuals demonstrated high consistency across sessions.

**Figure 9:**
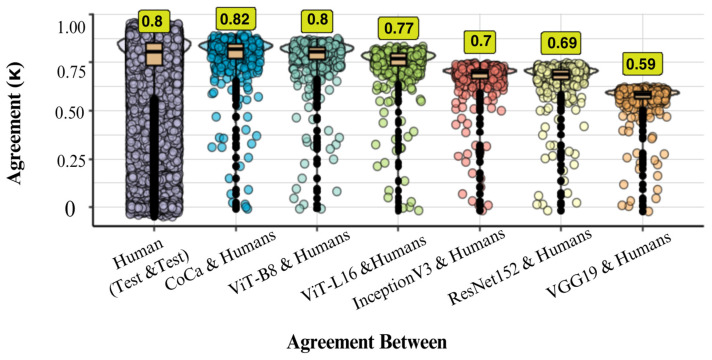
Distribution of overall agreement (unweighted Kappa-values) for human-human comparisons at 50 ms and human-DNN comparisons. The median Kappa values for each comparison group are emphasised in yellow boxes. Distributional information is presented as a box plot, a violin plot, and individual data points. The box plot showcases the median performance and interquartile range, with solid black circles indicating potential outliers identified using the interquartile range criterion.

Figure 9 shows that inter-rater agreement in the test session was also substantial but slightly lower than intra-rater agreement. This observation, combined with the distribution of Kappa values, which ranges widely—from negative values to those nearing the ideal agreement of 1—suggests that this difference may stem from human variability. The agreement between human participants and machine models varied significantly across models: CoCa and ViTs aligned most closely with human responses (0.77 < κ < 0.82), with ViT-B8 equalling human–human agreement and CoCa exceeding it, whereas DCNNs showed moderate to substantial alignment (0.59 < κ < 0.70). When comparing different types of models, Transformer-based models also exhibited the highest agreement with each other (0.89 < κ < 0.90), while VGG19 consistently showed the lowest agreement with both humans and other models (see Table 3).

#### 4.3.5. Results of Ordinal Discretisation

We used ordinal discretisation to simplify continuous data into qualitative patterns, highlighting the rank-order relationships between categories—specifically, in this case, the rank ordering of categorisation accuracy across different object categories. The analytical method of discretisation we used and its implementation are described in Appendix B.2 and Appendix B.3, respectively.

**Figure 10:**
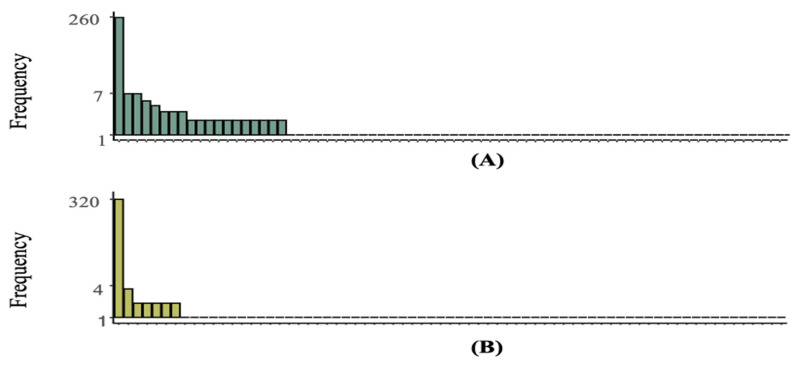
The bar graphs in Panels (**A**) (test) and (**B**) (retest) display the frequency of unique ordinal patterns identified in the test and retest sessions, respectively. In the Panels, the *y*-axis uses a logarithmic scale to better represent the wide range of unique patterns, ranked by the frequency of the observed patterns on the *x*-axis.

Following the discretisation of human data collected from test and retest sessions into an ordinal pattern framework based on nine performance categories, 103 unique patterns emerged in the test session at different frequencies (refer to Figure 10A), compared to 71 in the retest (refer to Figure 10B). This reduction indicates that, in the retest session, many individuals failed to produce their ordinal patterns from the test. Specifically, 60% of individuals maintained the same ordinal pattern across both sessions, while the remaining 40% demonstrated an inconsistency with their ordinal pattern in the retest session.

**Figure 11:**
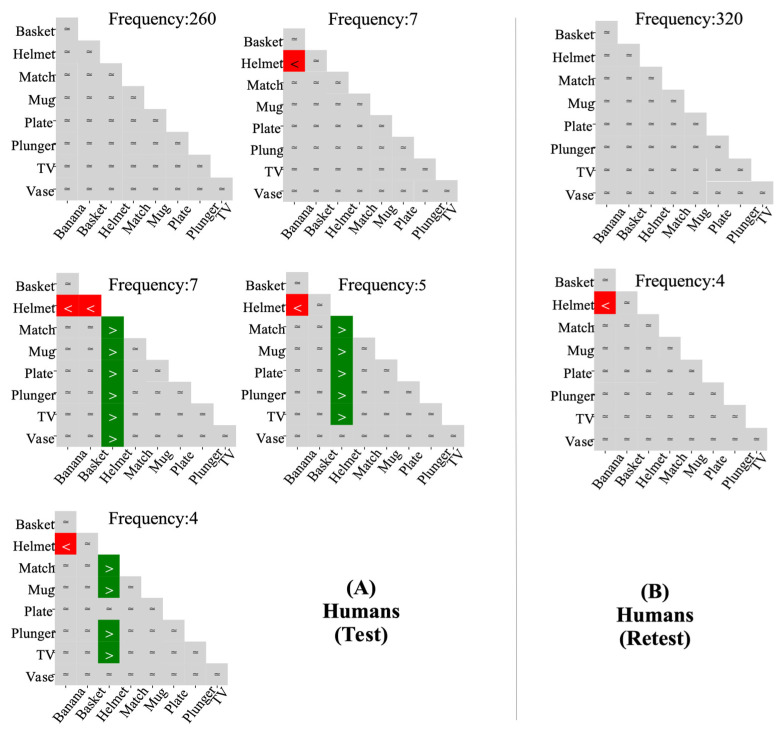
Panels (**A**,**B**) display the most frequently observed ordinal patterns with a frequency greater than 3 for the test and retest sessions. Panel (**A**) illustrates the top five most common ordinal patterns from the test session, while Panel (**B**) highlights the top two common patterns from the retest session. Each cell in a triangular matrix represents the relationship between two categories—reading from the row category to the column category—based on their proportion of correct responses out of 30 trials. The cells in the matrix represent one of three possible relationships: < (smaller; shown in red), > (larger; shown in green), or ≃ (approximately equal; shown in light grey). The frequency of each ordinal pattern, reflecting its occurrence within diverse subgroups, is noted above each pattern.

Notably, despite differences in the total number of heterogeneous patterns observed across sessions, the most common ordinal pattern remained the same in both sessions (as depicted in Figure 11); this implies that, although individuals may exhibit variability in their patterns across sessions, some patterns are prevalent enough to dominate the dataset. Furthermore, the observed decline in heterogeneous ordinal patterns appears to result from the increased frequency of this most common pattern in the retest session.

**Figure 12:**
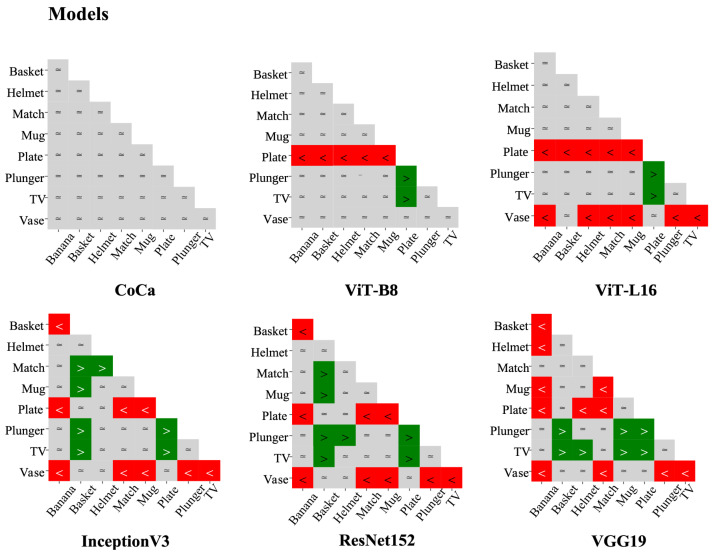
The observed qualitative ordinal patterns, including ordinal relationships between categories, are illustrated for each DNN model. Each cell in a triangular matrix represents the relationship between two categories—reading from the row category to the column category—based on their proportion of correct responses out of 30 trials. The cells in the matrix represent one of three possible relationships: < (smaller; shown in red), > (larger; shown in green), or ≃ (approximately equal; shown in light grey).

Figure 11 shows that the most common human ordinal pattern—equal performance across all categories—was observed in 65% of participants in the test session and 80% in the retest. Variations in this pattern primarily emerged in the components involving the Helmet category and its interactions with other categories. Notably, CoCa was the only model whose ordinal pattern showed equalities across all ordinal relationships among categories, mirroring the most frequently observed pattern in humans (see Figure 12). DCNNs exhibited the poorest alignment with human performance—performing better in easy categories but struggling significantly in more challenging ones—further highlighting their divergence from human patterns.

### 4.4. Experiment 3: Discussion

We compared human and machine performance in object recognition using both aggregate accuracy measures and individual-level pattern analysis. Overall, recent SOTA models—particularly the Vision Transformers (ViTs) and CoCa—matched or exceeded human accuracy and outperformed DCNNs. These models also showed greater alignment with human response patterns at the group level. Notably, CoCa not only achieved high accuracy but also reproduced the most common individual ordinal pattern observed in humans, showing alignment with human individual-level performance.

## 5. General Discussion

Over the past decade, advances in hardware, network architectures, and large-scale datasets such as ImageNet and JFT-300M have driven rapid progress in deep learning. These developments have led to claims that machine vision systems can match or surpass human-level performance (Dehghani et al., 2023; He et al., 2015; Krizhevsky et al., 2012; LeCun et al., 2015; Russakovsky et al., 2015; Zhu et al., 2017). However, such claims often rely on top-5 accuracy in closed-set tasks, use inconsistent category definitions, and fail to control for fundamental differences between human and machine vision.

More rigorous comparisons have addressed these limitations by aligning experimental conditions across the two systems. For example, Geirhos et al. (2021) tested humans and models on the same diverse and ecologically valid stimulus manipulations (e.g., noise, blur, style transfers) under brief, masked presentation to limit recurrent processing, with the results reanalysed by Dehghani et al. (2023). These studies show that while some models can match or exceed humans in average accuracy, they often fall short in replicating human-like generalisation, error patterns, and perceptual biases.

Recent work suggests that advanced architectures are narrowing the gap in OOD robustness and sometimes exhibit more human-like shape bias and error consistency (Geirhos et al., 2021; Jaini et al., 2023). Nonetheless, others argue that even the most accurate models still diverge from biological vision and fail to capture key aspects of human decision-making (Bowers et al., 2023; Fel et al., 2022).

Given these differing perspectives, this study compared a range of machine vision models—from traditional DCNNs to SOTA multimodal systems—against human performance. Following principles from comparative psychology and recommendations by Firestone (2020), similar constraints were imposed on both systems to ensure fairness. Using the well-controlled, ecologically valid ObjectNet dataset, we examined whether these models could achieve human-level accuracy and human-like classification behaviour under varying conditions. The results offer a nuanced understanding of the extent to which these models align with human vision, highlighting both their strengths and limitations.

To facilitate a fairer comparison, Experiment 1 adopted a 10-way categorisation approach for humans and fine-tuned machine models while reducing human stimulus presentation times (50 ms and 200 ms). Under these conditions, CoCa and Vision Transformers (ViTs) matched or exceeded human performance, whereas traditional DCNNs (excluding VGG19) performed comparably to humans at 50 ms. However, despite these improvements, models struggled with generalisation across challenging object categories, even after fine-tuning.

A potential explanation for the struggles in effectively generalising certain categories is the 3D viewpoint dependence and overreliance on surface characteristics in DNNs, such as texture and local details. Prior research has demonstrated that deep learning models, even the most recent SOTA models, struggle with viewpoint invariance and three-dimensional object representation (Alcorn et al., 2019; Borji & Itti, 2014; Cooper et al., 2021; Dong et al., 2023) and tend to classify objects based on textural patterns rather than global shape (Brendel & Bethge, 2019; Baker et al., 2018; Geirhos et al., 2019, 2021; Malhotra et al., 2022). This was particularly evident in this study, as “plates” emerged as the most challenging category—likely due to the high variation in 2D appearance under rotation and the model’s tendency to over-rely on local texture and colour features rather than global object structure. In contrast, human observers rely more on object-centred, three-dimensional shape representations, enabling greater robustness across varying orientations (Erdogan & Jacobs, 2017). Although multimodal foundational models such as CLIP have shown improved shape bias compared to earlier CNNs, they still rely more on texture than human observers (Geirhos et al., 2021). Similarly, CoCa, as a multimodal foundation model, outperformed other DNNs tested in this study across these challenging categories.

Following this, the motivation for designing Experiment 2 stemmed from the initial intuition that fine-tuning was a reasonable engineering approach to the N-way categorisation procedure in Experiment 1, potentially giving machine systems an advantage over humans. Experiment 2 compared human performance under a free-naming paradigm with machine models evaluated on their original ImageNet-trained output layers without additional fine-tuning. The first assumption was that human performance would remain relatively unaffected during the transition from N-way categorisation to free-naming, while DNN performance would decline due to the loss of fine-tuning benefits. Consistent with the first assumption, machine models showed a significant decline in performance after losing the advantage of fine-tuning compared to their performance in Experiment 1. Humans also experienced a decline in accuracy when transitioning to free-naming in the 50 ms condition; however, this decline was smaller than that of the DNNs, indicating that biological vision is more robust and flexible, whereas deep learning models rely on structured classification tasks for optimal performance.

Since the models tested in this study were designed for object recognition rather than object detection, bounding boxes were introduced to remove most of the background surrounding the objects and assess whether both humans and machines perform better when objects are presented in isolation versus within full-scene images with intact background complexity. Machine models showed a clear improvement in accuracy when objects were presented in isolation, consistent with findings that background removal yields a 20–30% increase in accuracy on ObjectNet (Borji, 2021). While humans also exhibited a decline in accuracy under full-scene conditions, the magnitude of this decline was less pronounced than for machine models. The results also indicated that, when detection is not a factor, humans consistently outperformed machines in the isolated object condition, underscoring humans’ superior object-recognition abilities.

The declines in accuracy observed for both humans and models under the full-image condition suggest that contextual factors—particularly inconsistencies between objects and background scenes—influence recognition performance. Previous research has shown that deep learning models often rely on background cues as shortcuts for object classification, which can lead to errors when objects appear in atypical or cluttered environments (Bell et al., 2016; Beery et al., 2018). For humans, contextual information plays a dual role: it can facilitate recognition when consistent with the object but hinder it when incongruent (Oliva & Torralba, 2007).

To further examine the influence of contextual background on recognition accuracy, images were categorised as neutral (minimal or ambiguous backgrounds), congruent (typical real-world settings), or incongruent (atypical settings, e.g., a plate on a bed). In the isolated-object condition, most images were neutral (97.8%) with very few incongruent examples (2.2%). In contrast, the full-image condition contained a higher proportion of incongruent contexts (43.7%), one-third neutral contexts (37.8%), and fewer congruent contexts (18.5%). This higher proportion of incongruent backgrounds likely contributed to the decline in performance observed for both humans and models in the full-image condition.

Experiment 3 expanded on Experiments 1 and 2 by examining individual differences in human categorisation performance and addressing a key limitation of previous experiments, as traditional group-level analyses often obscure variability in human performance (Estes, 1956). To address this limitation, a data discretisation method was used to identify category-level performance patterns based on ordinal relationships between category performances at the individual level, with a large participant pool tested under the 50 ms condition. The results showed that CoCa and ViTs performed comparably to, or better than, DCNNs and humans at the group-level performance. However, at the individual level, CoCa was the only model whose ordinal pattern mirrored the most common human pattern. Specifically, the ordinal pattern of CoCa exhibited equal ordinal relationships across all categories, replicating the equality-driven structure observed in the most common human pattern. This suggests that multimodal models that integrate language priors more closely approximate human object recognition. In contrast, the ordinal patterns of other models, such as ViTs and DCNNs, showed no overlap with human patterns, as they included ordinal relationship components between category pairs that were not observed in humans.

This study presents key findings regarding the alignment between human and machine categorisation behaviour, highlighting both areas of agreement and notable discrepancies. Across all experiments, CoCa exhibited the highest agreement with human responses, followed by ViTs, while traditional DCNNs demonstrated the lowest alignment. Notably, as model accuracy improved, agreement between human and machine responses also increased. However, discrepancies became particularly evident when analysing misclassification patterns. Even when both humans and models misclassified the same stimuli, their incorrect responses often differed, a trend most pronounced in the full-image condition of Experiment 2. A key factor contributing to these differences is the method used to collect responses. In Experiment 2, human participants provided free-form names, whereas models were selected from a fixed set of 1000 object classes. In contrast, Experiment 1 limited both humans and models to a fixed set of 10 object classes, increasing the likelihood of direct response matching. The larger response pool in Experiment 2 reduced the probability that both systems would provide the same incorrect answer, thereby decreasing apparent agreement. This methodological difference highlights an intriguing area for future research in comparing human and machine errors: even when models achieve comparable accuracy to humans, the nature of their mistakes can differ significantly. This finding aligns with prior research on error consistency: Geirhos et al. (2021) showed that deep neural networks, even those trained for OOD robustness, still process images differently from human observers, suggesting that machines may rely on fundamentally different feature representations than humans when classifying objects, despite similar overall performance.

### 5.1. Explaining Variability in Overall Model Accuracy Across Experiments

Model performance varied substantially across experiments, with the poorest results in Experiment 2 and higher accuracy in Experiments 1 and 3. For example, CoCa achieved approximately 70% accuracy in Experiment 2, compared to 92% in both Experiments 1 and 3. Although this variability might initially appear inconsistent, it can be explained by several key experimental and computational factors that differed across conditions.

In Experiment 2, models were evaluated using their original ImageNet-trained output layers across 1000 classes, without fine-tuning or task-specific zero-shot classification. Humans performed a free-naming task, while models generated predictions from the full 1000-class space—likely contributing to the performance gap. In contrast, experiments 1 and 3 used 10-way and 9-way forced-choice tasks, respectively, with models fine-tuned or adapted to the specific categories. Fine-tuning markedly improved accuracy, particularly for CoCa and ViTs; both humans and the top-performing models achieved similar overall accuracies in both experiments (86% for humans, 92% for CoCa, 90% for ViT-B8, and ~87% for ViT-L16).

However, a key difference emerged in the statistical interpretation of these results. Although models’ performance in Experiment 1 was numerically higher than that of humans, Bayesian analysis did not provide conclusive evidence for a difference. In Experiment 3, however, the same models formally outperformed humans—likely due to the substantial difference in sample size—25 participants in Experiment 1 versus 398 in Experiment 3. Collectively, performance differences across experiments can be explained by interacting factors: task type (free-naming vs. forced-choice), model configuration (pretrained vs. fine-tuned), stimulus duration, and masking. For optimising model performance while maintaining fairness, conditions similar to Experiments 1 and 3 are preferable—limited label sets, fine-tuning, brief stimulus presentations, and masking. For assessing generalisation, Experiment 2 provides a stricter benchmark by testing models in their original state against human free-naming. Future work could align conditions further by testing humans on 1000-way classification tasks.

### 5.2. Future Research Directions

The findings of this study suggest several key directions for future research. Future efforts should investigate how different training paradigms impact the generalisation abilities of machine models. One important consideration is that models in this study were trained on vastly different datasets. The DCNNs were pre-trained on the ImageNet dataset, which contains 1000 classes and 1.3 million images (Deng et al., 2009). In contrast, ViTs were pre-trained on the much larger ImageNet-21K dataset, comprising 14 million images and 21,000 categories, before fine-tuning on the standard ILSVRC-2012 ImageNet dataset. Meanwhile, CoCa was pre-trained on an even larger dataset—13 billion image-text pairs from LAION-2B (Schuhmann et al., 2022). These discrepancies in the training data may introduce variables that could affect comparisons of model performance. Future studies should control for these differences by training models on the same dataset or systematically varying dataset size and composition to assess their impact on generalisation.

The findings in Experiment 2 also underscore the importance of separating detection and classification tasks when evaluating artificial recognition models to ensure fair comparisons with human performance, particularly for tasks requiring adaptability to real-world conditions. By comparing human and machine recognition under isolated and full-image conditions, this study identifies areas where current artificial vision models fall short and offers benchmarks for their improvement. For instance, the comparable performance of CoCa with humans at 50 ms in the full-image condition suggests that integrating language capabilities may partially compensate for the absence of localisation mechanisms in existing vision models. Borji (2021) also suggested that future advancements in artificial recognition systems could benefit from integrating detection and classification capabilities or adopting alternative evaluation methods, such as multi-label prediction accuracy, to better reflect performance in naturalistic settings.

Another important direction for future work is that, although overall accuracy provides a useful benchmark, more fine-grained comparisons—such as confusion–matrix–based analyses or representational similarity approaches—may offer additional insight into the extent to which models and humans show similar categorisation structures. In the present study, near-ceiling performance in several conditions limited the interpretability of such analyses, as the number of informative errors was small. Related representational similarity analyses were explored in earlier work (Kul, 2025), yet yielded inconsistent results across experiments, highlighting known limitations of using correlation-based approaches as stand-alone measures for evaluating human–machine alignment (Popal et al., 2019). The modulation effect (Kriegeskorte et al., 2009) suggests that RSA scores depend heavily on dataset structure, and the mimic effect (Dujmović et al., 2022) indicates that models can appear to share representational structures with humans due to second-order dataset biases rather than to truly similar feature-processing mechanisms. Future research may explore alternative representational analysis techniques, such as encoding models, pattern component analysis, or multivariate dependence methods, as suggested by Popal et al. (2019), to more rigorously validate RSA findings.

Lastly, a novel contribution of this study is the comparison of various machine learning models with human categorisation performance while accounting for individual differences in human categorisation behaviour. Future research could benefit from a more systematic approach to measuring heterogeneity in human categorisation, such as calculating the g-distance for each machine model. This metric assesses the extent to which a model’s range of behaviours overlaps with the diversity of human responses, thereby providing a formal measure of model adequacy based on the degree of shared behavioural variability (see Dome & Wills, 2025). By comparing model-generated categorisation patterns with those observed in human participants, g-distance provides a structured approach to evaluating how closely a model replicates human-like variability in object classification.

Applying this approach within a formal categorisation modelling framework would enable researchers to quantify the alignment between model outputs and individual-level variation in human behaviour, rather than relying solely on group-level comparisons. This could provide deeper insights into whether certain models reflect human heterogeneity in object classification performance, particularly in challenging object recognition tasks.

## 6. Conclusions

This study highlights the remarkable progress made in artificial vision, with recent machine learning models approaching and sometimes exceeding human-level performance under optimised conditions. However, the major challenge remains: while deep learning models, particularly recent advancements like CoCa and ViTs, have shown improved performance and better alignment with human response patterns—closing the gap with human performance in terms of robustness on Out-of-Distribution (OOD) image sets—their performance on the most challenging categories remained significantly lower than human observers. This indicates that even the most recent advanced machine models continue to struggle with generalisation, particularly when dealing with the variability and complexity of specific real-world object categories. This limitation is particularly noticeable in scenarios without fine-tuning or task-specific zero-shot classification.

Notably, CoCa, a multimodal image-text foundational model not designed for object recognition, demonstrated impressive zero-shot image classification performance. CoCa showed promise in enhancing robustness and performance on challenging categories, outperforming all DNNs tested in this study. Moreover, CoCa aligned with individual-level human performance, replicating the most common ordinal patterns observed across human participants. This suggests that integrating language into vision models—coupled with architectural advancements such as attention mechanisms, training on vast datasets through unsupervised learning, and incorporating training objectives like contrastive and captioning losses—enhances their robustness and improves generalisation on OOD benchmark sets. Previous studies also support the idea that incorporating language into vision models enhances their ability to generalise, aligning their internal representations more closely with human similarity judgments (Marjieh et al., 2022; Muttenthaler et al., 2022; Wang et al., 2023).

## Figures and Tables

**Table 1:** The overall agreement (Kappa-values) between DNNs.

	CoCa	ViT-B8	ViT-L16	ResNet152	InceptionV3
ViT-B8	0.87				
ViT-L16	0.87	0.88			
ResNet152	0.76	0.78	0.78		
InceptionV3	0.74	0.75	0.75	0.74	
VGG19	0.66	0.67	0.68	0.67	0.66

**Table 2:** The overall agreement (Kappa values) between DNNs under isolated objects and full-image conditions.

Condition	CoCa	ViT-B8	ViT-L16	ResNet152	InceptionV3
Isolated					
ViT-B8	0.65				
ViT-L16	0.63	0.76			
ResNet152	0.46	0.54	0.52		
InceptionV3	0.43	0.46	0.42	0.51	
VGG19	0.28	0.34	0.34	0.32	0.39
Full					
ViT-B8	0.62				
ViT-L16	0.55	0.68			
ResNet152	0.50	0.53	0.50		
InceptionV3	0.39	0.43	0.40	0.45	
VGG19	0.25	0.30	0.31	0.39	0.32

**Table 3:** The overall agreement (Kappa-values) between DNNs.

	CoCa	ViT-B8	ViT-L16	ResNet152	InceptionV3
ViT-B8	0.89				
ViT-L16	0.90	0.90			
ResNet152	0.77	0.79	0.79		
InceptionV3	0.73	0.76	0.76	0.72	
VGG19	0.65	0.67	0.69	0.63	0.70

**Figure A1:**
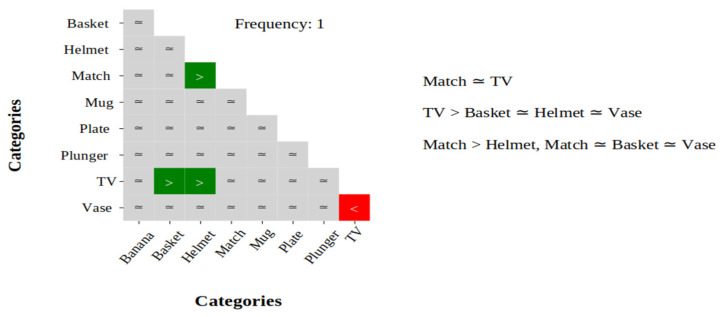
One of the rare ordinal patterns observed in the human test session, consisting of 36 components, was observed in only one participant. Each component of the triangular matrix represents the relationship between two categories—read from the row category to the column category—based on their proportion of correct responses out of 30 trials. The cells in the matrix indicate one of three possible relationships: < (smaller; shown in red), > (larger; shown in green), or ≃ (approximately equal; shown in light grey). A subset of the relationships from this pattern is also represented as a single ordered set next to the matrix.

## Data Availability

The human data, along with all related materials and analysis code, are available at [https://github.com/gokcekul/object-recognition-paper, accessed on 27 October 2025]. However, human data from Experiment 3 are not publicly shared, as they are reserved for future analyses and publications.

## Supplementary Materials

The following supporting information can be downloaded at: https://www.mdpi.com/article/10.3390/bs16010109/s1, Figure S1: Accuracy of humans and the artificial vision systems for easy object categories across isolated objects and full-image conditions. Human 50 ms and Human (self-pace) are the accuracies of each individual at different stimuli presentation times, as 50 ms and self-paced viewing; CoCa, ViT-B8, ViT-L16, ResNet152, InceptionV3 and VGG19 are the artificial vision systems. The box plot showcases the median performance and interquartile range of human data, with solid black circles indicating potential outliers identified using the interquartile range criterion. Significance lines indicate comparisons between human and model accuracies: comparisons to Humans (self-pace) are shown above the boxplots, and comparisons to Humans 50 ms are shown below. Different signifiers indicate varying levels of Bayesian evidence for differences, with * indicating BF > 3, ** indicating BF > 10, and *** indicating BF > 100; Figure S2: Accuracy of humans and the artificial vision systems for moderate object categories across isolated objects and full-image conditions. Conventions are identical to Figure S1; Figure S3: Accuracy of humans and the artificial vision systems by challenging object categories across isolated objects and full-image conditions. Conventions are identical to Figure S1; Figure S4: Accuracy of humans and the artificial vision systems by easy object categories. “Human 50 ms” denotes the accuracy of individuals during the test session at a 50 ms presentation time.; CoCa, ViT-B8, ViT-L16, ResNet152, InceptionV3 and VGG19 are the artificial vision systems. The box plot showcases the mean performance and interquartile range of human data, with solid black circles indicating potential outliers identified using the interquartile range criterion. Significance lines indicate comparisons between human and model accuracies: advantages favouring humans are shown above the box plots, while those favouring each DNN are shown below. Different signifiers indicate varying levels of Bayesian evidence for differences, with * indicating BF > 3, ** indicating BF > 10, and *** indicating BF > 100; Figure S5: Accuracy of humans and the artificial vision systems by moderate object categories. Conventions are identical to Figure S4; Figure S6: Accuracy of humans and artificial vision systems by challenging object categories. Conventions are identical to Figure S4.

## Abbreviations

The following abbreviations are used in this manuscript:

AI	Artificial Intelligence
BF	Bayes Factor
DNNs	Deep Neural Networks
DCNNs	Deep Convolutional Neural Networks
IID	Independent and Identically Distributed
OOD	Out-of-Distribution
SOTA	State-of-the-Art
ViTs	Vision Transformers

## Appendix A. Model Selection

To explore how different training objectives, datasets, and architectures affect machine performance, we selected various DNNs that represent key developments in the history of deep learning systems. We will elaborate on the details of each selected model, presented chronologically according to their emergence.

Firstly, we chose three DCNNs pre-trained in a supervised manner on the ImageNet dataset, which contains 1000 classes and 1.3 million images. These models include VGG19 (Simonyan & Zisserman, 2014), ResNet152 (He et al., 2016), and InceptionV3 (Szegedy et al., 2016), all accessed through the PyTorch Image Models (TIMM) library (Version 0.9.12), a Python library for image classification.

Next, Vision Transformers (ViTs) (Dosovitskiy et al., 2020) were selected to represent a significant architectural shift from convolutional networks to models that utilise pure self-attention. Two ImageNet-trained ViT models were obtained from the TIMM library (Wightman, 2019)—specifically, we used “vit_base_patch8_224” and “vit_large_patch16_224”. The models were pre-trained on the larger ImageNet-21K dataset (Deng et al., 2009)—14M images with 21K classes—before they were fine-tuned on the “standard” ILSVRC-2012 ImageNet dataset with 1.3M images and 1K classes. They are referred to as ViT-B8 and ViT-L16 throughout the thesis.

Finally, we included CoCa—an image-text foundational model with self-supervised training on a large image-text dataset —reflecting the latest advancements in the field. We utilised the CoCa (Yu et al., 2022) implementation from the OpenCLIP GitHub repository (Ilharco et al., 2021). The best-performing model has not yet been made publicly available, so we used CoCa-ViT-L-14, pretrained on 13 billion samples from the LAION-2B public dataset, suitable for training large image-text models (Schuhmann et al., 2022). Throughout the manuscript, “CoCa” refers to CoCa-ViT-L-14.

## Appendix B

### Appendix B.1. Analytical Methods

#### Accuracy Coding and Response Correction in the Free-Naming Task

The free-naming paradigm determined accuracy by comparing participants’ typed responses to the actual object class. Given the open-ended nature of this task, a cleaning and correction procedure was implemented to analyse participants’ responses.

First, all responses were pre-processed using R (Version 4.3.2; R Core Team, 2023) by removing leading and trailing spaces and converting them to lowercase to ensure consistency with the ObjectNet labels. Following this, the data from the first participant were manually inspected to identify common issues. Spelling and typing errors, such as “plnger” and “plungfer,” were corrected to “plunger.” Synonyms and alternative spellings were standardised; for example, “matchstick” was simplified to “match” and “television” to “tv”.

Multi-object references in responses where images included multiple objects and the response mentioned both, such as “vase and flowers” or “vase on the sofa”, were simplified to “vase”. Corrections were applied if the response included the correct ObjectNet label for the image to align with the target category. This adjustment accounted for images where the primary object of interest (e.g., “vase”) co-appeared with other elements like flowers or background details.

Responses containing more than one word, including descriptive terms and contextual information, were simplified to match the target label (e.g., “a lit match” was corrected to “match” or “blue vase”, and “vase of flowers” was corrected to “vase”). Nonessential elements, including indefinite articles such as “a match,” plural forms like “matches,” and punctuation marks (e.g., “match]” or “plate#”), were removed to ensure consistency with the expected labels.

Based on initial corrections identified in the first participant’s data, programmatic rules were developed in R to automate the cleaning process. These rules were then applied to subsequent participants’ responses to facilitate further corrections. After each automated pass, any remaining inaccuracies in subsequent responses were manually reviewed and incorporated into the programmatic correction rules. This iterative process—alternating automated correction with manual inspection—was repeated for each participant, ensuring comprehensive coverage of errors and minimising manual intervention in subsequent data processing.

After corrections, responses were coded as correct if they contained the correct ObjectNet label and incorrect otherwise. By combining manual validation with programmatic rules, the correction process ensured fairness in evaluating participants’ performance and comparability between human responses and model outputs.

### Appendix B.2. Discretisation

The method of discretisation we used to analyse our data is ordinal discretisation, based on the work of Dome and Wills (2025). Ordinal discretisation simplifies continuous data into qualitative patterns, highlighting the rank-order relationships between categories—specifically, in this case, the rank ordering of categorisation accuracy across different object categories. By focusing on these ordinal patterns rather than raw numerical differences, this approach captures general trends or patterns in the data.

To illustrate the core principles of the method, consider a simplified example involving three categories—Vase, Helmet, and Banana—instead of the nine categories analysed in the experiment. Suppose participants’ performances were evaluated based on 90 images, with 30 images per category: Vase, Helmet, and Banana. The number of correct responses recorded was 19 for Vase, 17 for Helmet, and 28 for Banana. Consequently, the proportional accuracies for these categories were 0.63, 0.57, and 0.93, respectively. A clear ordinal pattern was established, displaying a strong ranking order (e.g., Banana > Vase > Helmet), with no ties present. Alternatively, a weak order allows for an equal relationship, suggesting general rankings within equivalence classes (e.g., Banana > Vase = Helmet), acknowledging that measurement errors can have a significant impact on human data (see Pitt et al., 2006). Traditionally, experimental psychologists have concentrated on finding substantial evidence for inequalities through methods such as null hypothesis significance testing or within a Bayesian framework. Approximate equality (≃) was defined by the threshold of difference necessary to consider that difference reliable. Thus, in both traditional and Bayesian contexts, one can view ordinal patterns as comprising inequalities (<, >) and approximate equalities (≃).

In this experiment, an ordinal pattern of each individual’s performance on nine object categories, denoted as N (which represents the number of components), is formed by 36 unique pairwise relationships between categories. The number of pairwise relationships (M) is derived from the formula M = N(N − 1)/2, highlighting the combinatorial relationships between the categories. To systematically portray these relationships, we used triangular matrices to represent these relationships formally, capturing the intricate interplay and all pairwise comparisons among categories, as the recommended method of representing the outcome of ordinal discretisation by Dome and Wills (2025). This is because not all triangular matrices can be succinctly represented by a single ordered set due to the non-transitive relationships in patterns. For instance, in a situation where the relationships follow a non-transitive pattern: Banana ≃ Vase ≃ Helmet, yet Banana > Helmet. In such cases, a simple linear ranking fails to capture the complexity of these relationships. Consequently, the utility of the triangular matrix—as illustrated in Figure A1—provides a formalisation that effectively represents complex patterns that cannot be expressed compactly in ordered sets.

### Appendix B.3. Implementation and Results of Ordinal Discretisation

We discretised human data using a method similar to that outlined by Dome and Wills (2025), which employs a Bayesian version of a difference-of-proportions test to establish ordinal relationships among response proportions for different object categories. While a frequentist version of the difference-of-proportions test was employed to achieve the same goal of discretising ordinal relationships among the categories, we adopted a similar threshold-based framework. This involved classifying response proportions into three categories based on a predefined threshold: greater than (>), less than (<), and approximately equal (≃). If the difference exceeds this threshold, it is indicated with an inequality (“>” or “<”); if it reflects equality or a smaller difference, it is approximately equal (≃).

In the frequentist version of a difference-of-proportions test approach, we used the “prop.test” function in RStudio (Version 2023.6.1.524) to perform a proportion test to assess all possible combinations of response counts from two categories, each generated from a total of 30 trials. Using this method, we calculated *p*-values for each pairwise comparison of proportions. After computing *p*-values for all pairwise comparisons, a threshold was determined to declare a notable difference that would signify a meaningful discrepancy. This threshold was derived from all possible observable results of the pairwise comparisons and is in line with the Bonferroni-corrected alpha level. Bonferroni correction was applied to account for multiple comparisons. This conservative adjustment ensures that the probability of detecting a false positive (Type I error) across multiple tests remains within a chosen significance level (α = 0.05). The Bonferroni-adjusted alpha level was calculated as follows:

(A1) $$\mathrm{Bonferroni}\text{-}\mathrm{corrected}\;\alpha =\frac{\alpha }{n_{\mathrm{categories}}\frac{\left(n_{\mathrm{categories}}-1\right)}{2}}$$

where *n_categories_* is the total number of categories, this adjustment aids in maintaining the overall confidence level while performing multiple pairwise tests. The Bonferroni-corrected *p*-value threshold is established when the difference in response proportions reaches 0.3. For this analysis, we opted for this threshold, which means that a comparison will only be regarded as statistically significant if the difference in proportions between the two categories exceeds 0.3. This threshold ensured that only robust and substantial differences were treated as ordinal distinctions.

This methodological choice was made for both practical and statistical reasons. Unlike the frequentist framework, the Bayesian method used by Dome and Wills does not offer a direct analogue for multiple-comparison correction, and while advanced Bayesian techniques (e.g., hierarchical models) are available, they would have required additional model development beyond the scope of this project. By contrast, the frequentist approach—with Bonferroni correction—offered a well-established approach suitable for the larger comparison set in this study. For comparison, Dome and Wills (2025) used only five categories, resulting in 10 comparisons (one anchored to a fixed value of 0.5), making their analysis less vulnerable to the risk of false positives arising from multiple comparisons.

Note that while the Bonferroni correction provides robust control over Type I error rates, it is known to be conservative. Consequently, this approach may reduce the sensitivity of the analysis in detecting more subtle differences in categorisation performance and could lead to an underestimation of the true number of distinct patterns. However, this conservatism also increases confidence that the identified ordinal patterns are based on reliable distinctions in participant responses. Overall, the frequentist implementation provided a practical and defensible solution under the constraints of this project. Future work may benefit from exploring fully Bayesian discretisation approaches, such as hierarchical models using Dirichlet process priors to model latent patterns in individual differences (Navarro et al., 2006), to improve sensitivity without sacrificing robustness.

For each participant, we calculated performance metrics (proportion of correct responses out of 30 responses) for each of the nine categories. Then, these performance metrics were transformed into ordinal relationships (<, >, ≃) that reflect inequality and approximate equality, and encoded in a triangular matrix to represent ordinal relationships between categories. Based on the difference in the proportion of correct responses of each pair of categories, the difference was expressed as an inequality (> or <) if it exceeded the predefined threshold of 0.3; any differences in proportions less than or equal to 0.3 were expressed as approximately equal (≃). The categorisation performance of each DNN was discretised using the same threshold as in the human data, and the ordinal relationships between categories were represented in a triangular matrix as human ordinal patterns. By discretising the data in this way, we identified common and rare ordinal patterns across humans and examined whether these patterns aligned with any of those produced by artificial vision models.
