# Supplementary material for: The Comparison of Human and Machine Performance in Object Recognition
Source: Behav Sci (Basel). 2026 Jan 13;16(1):109. doi: 10.3390/bs16010109 (PMC12837923; doi:10.3390/bs16010109)
Supplement: Supplementary file 1 [file behavsci-16-00109-s001.zip › behavsci-3982873-supplementary.pdf]

Supplementary Materials

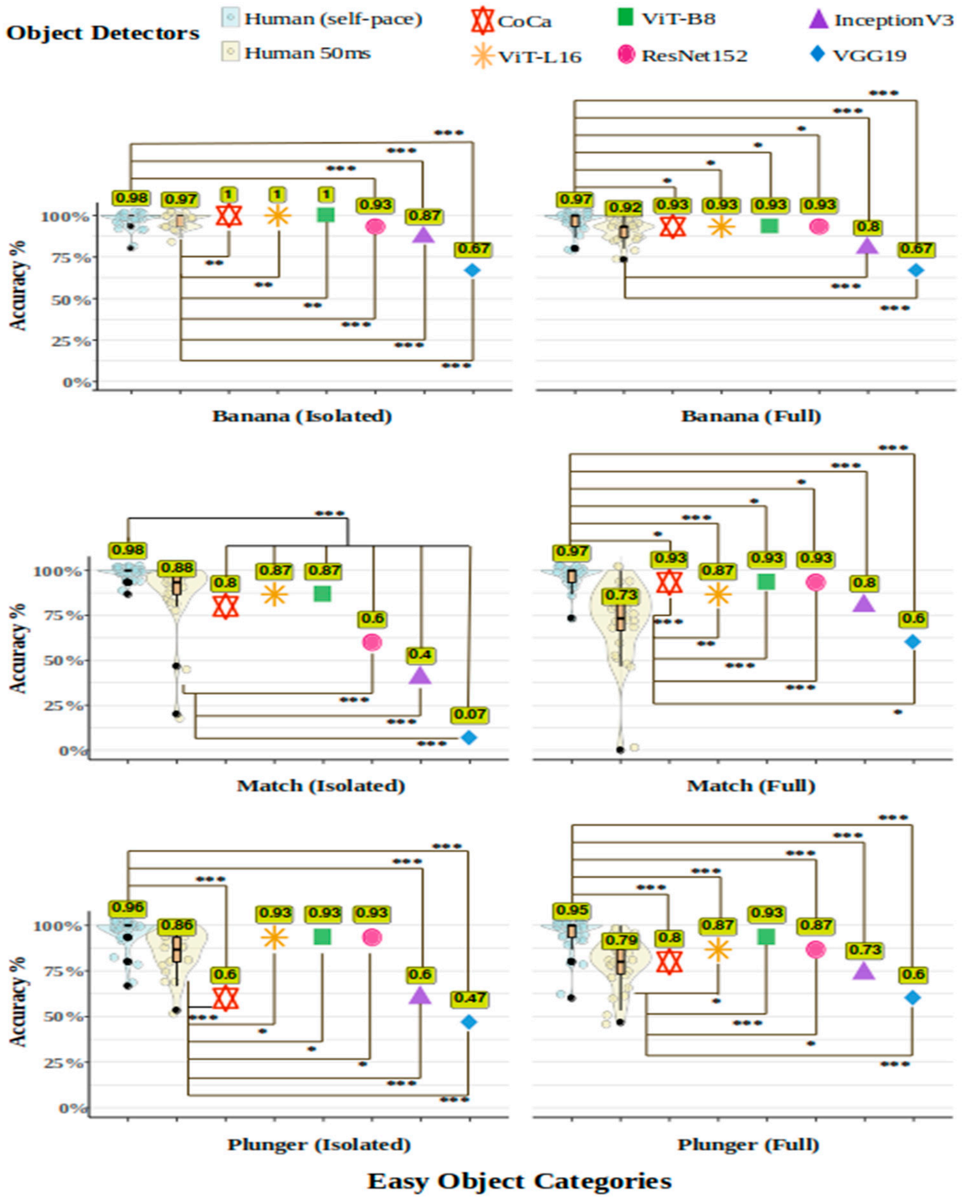

Figure S1. Accuracy of humans and the artificial vision systems for easy object categories across isolated objects and full-image conditions. Human 50ms and Human (self-pace) are the accuracies of each individual at different stimulus presentation times, as 50ms and self-paced viewing; CoCa, ViT-B8, ViT-L16, ResNet152, InceptionV3 and VGG19 are the artificial vision systems. The box plot showcases the median performance and interquartile range of human data, with solid black circles indicating potential outliers identified using the interquartile range criterion. Significance lines indicate comparisons between human and model accuracies: comparisons to Humans (self-pace) are shown above the boxplots, and comparisons to Humans 50ms are shown below. Different signifiers indicate varying levels of Bayesian evidence for differences, with * indicating BF > 3, ** indicating BF > 10, and *** indicating BF > 100.

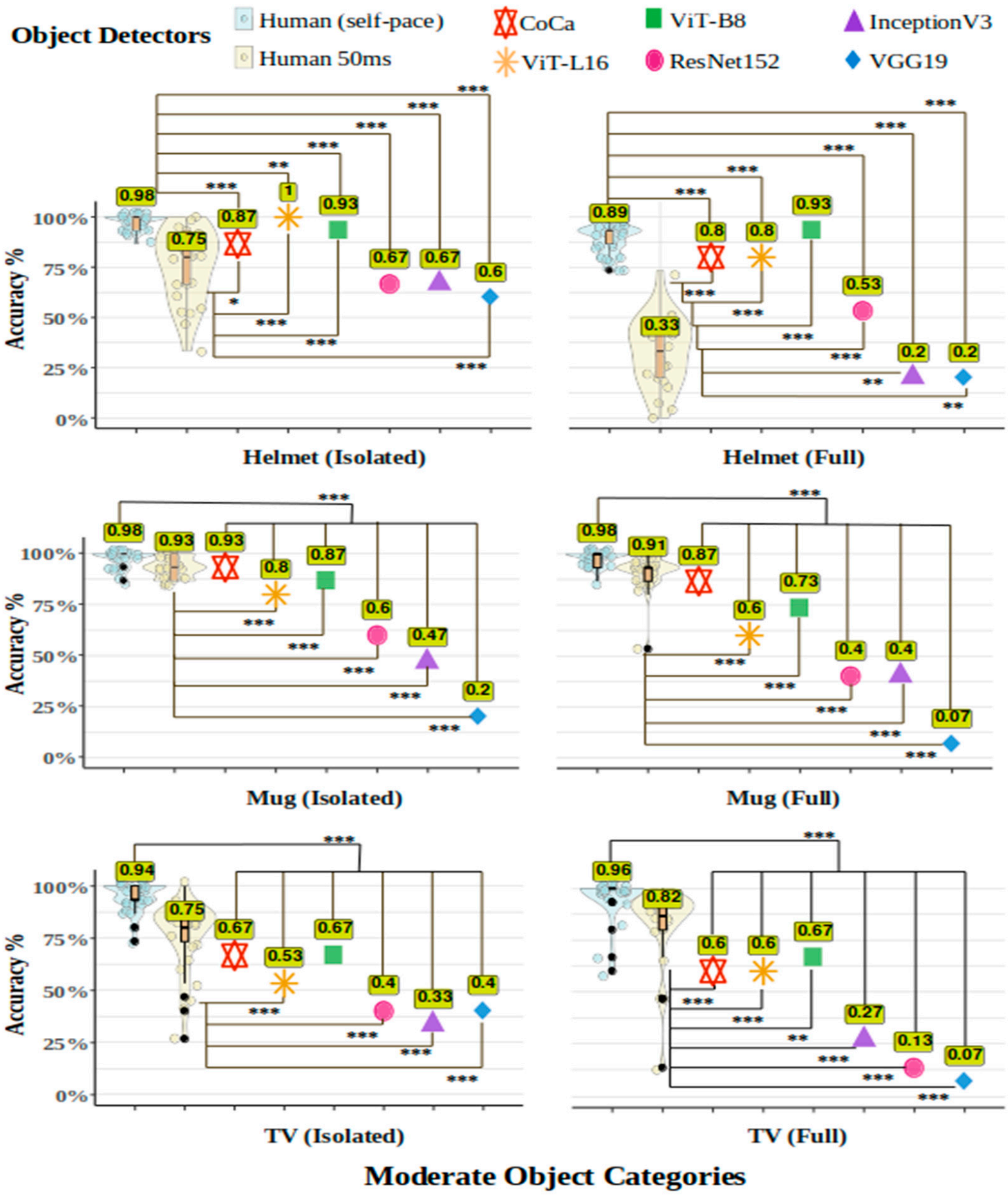

Figure S2. Accuracy of humans and the artificial vision systems for moderate object categories across isolated objects and full-image conditions. Human 50ms and Human (self-pace) are the accuracies of each individual at different stimuli presentation times as 50ms and self-paced viewing; CoCa, ViT-B8, ViT-L16, ResNet152, InceptionV3 and VGG19 are the artificial vision systems. The box plot showcases the median performance and interquartile range of human data, with solid black circles indicating potential outliers identified using the interquartile range criterion. Significance lines indicate comparisons between human and model accuracies: comparisons to Humans (self-pace) are shown above the boxplots, and comparisons to Humans 50ms are shown below. Different signifiers indicate varying levels of Bayesian evidence for differences, with * indicating BF > 3, ** indicating BF > 10, and *** indicating BF > 100.

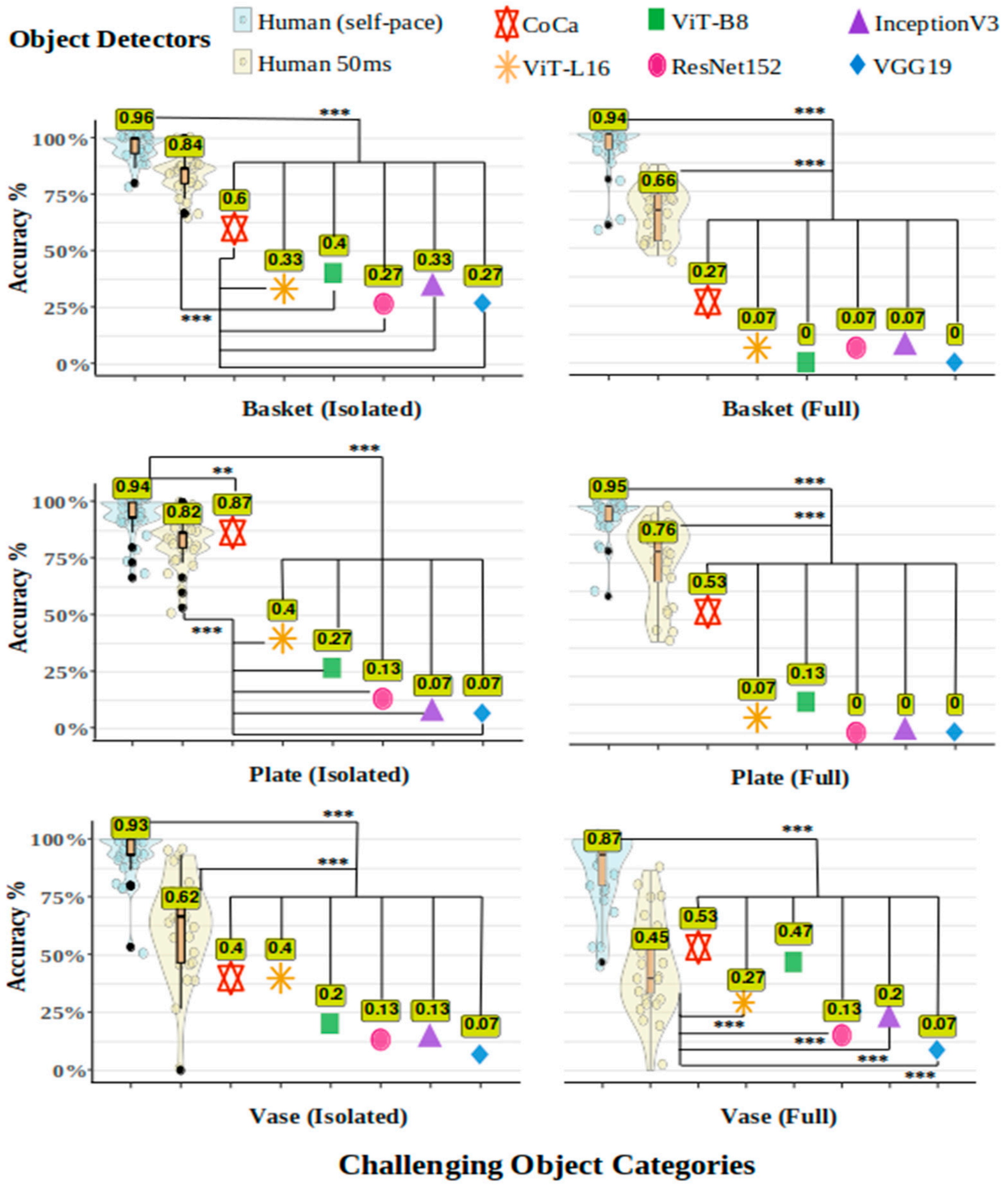

Figure S3. Accuracy of humans and the artificial vision systems by challenging object categories across isolated objects and full-image conditions. Human 50ms and Human (self-pace) are the accuracies of each individual at different stimuli presentation times as 50ms and self-paced viewing; CoCa, ViT-B8, ViT-L16, ResNet152, InceptionV3 and VGG19 are the artificial vision systems. The box plot showcases the median performance and interquartile range of human data, with solid black circles indicating potential outliers identified using the interquartile range criterion. Significance lines indicate comparisons between human and model accuracies: comparisons to Humans (self-pace) are shown above the boxplots, and comparisons to Humans 50ms are shown below. Different signifiers indicate varying levels of Bayesian evidence for differences, with * indicating BF > 3, ** indicating BF > 10, and *** indicating BF > 100.

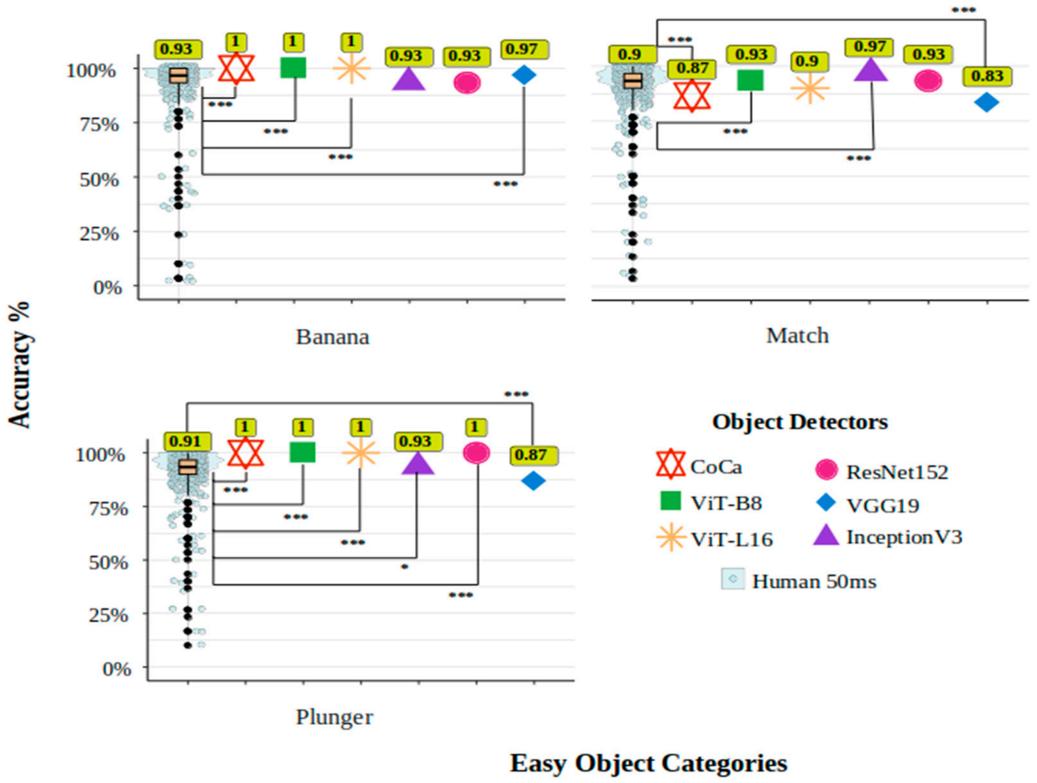

Figure S4. Accuracy of humans and the artificial vision systems by easy object categories. "Human 50ms" denotes the accuracy of individuals during the test session at a 50ms presentation time.; CoCa, ViT-B8, ViT-L16, ResNet152, InceptionV3 and VGG19 are the artificial vision systems. The box plot showcases the mean performance and interquartile range of human data, with solid black circles indicating potential outliers identified using the interquartile range criterion. Significance lines indicate comparisons between human and model accuracies: advantages favouring humans are shown above the box plots, while those favouring each DNN are shown below. Different signifiers indicate varying levels of Bayesian evidence for differences, with * indicating BF > 3, ** indicating BF > 10, and *** indicating BF > 100.

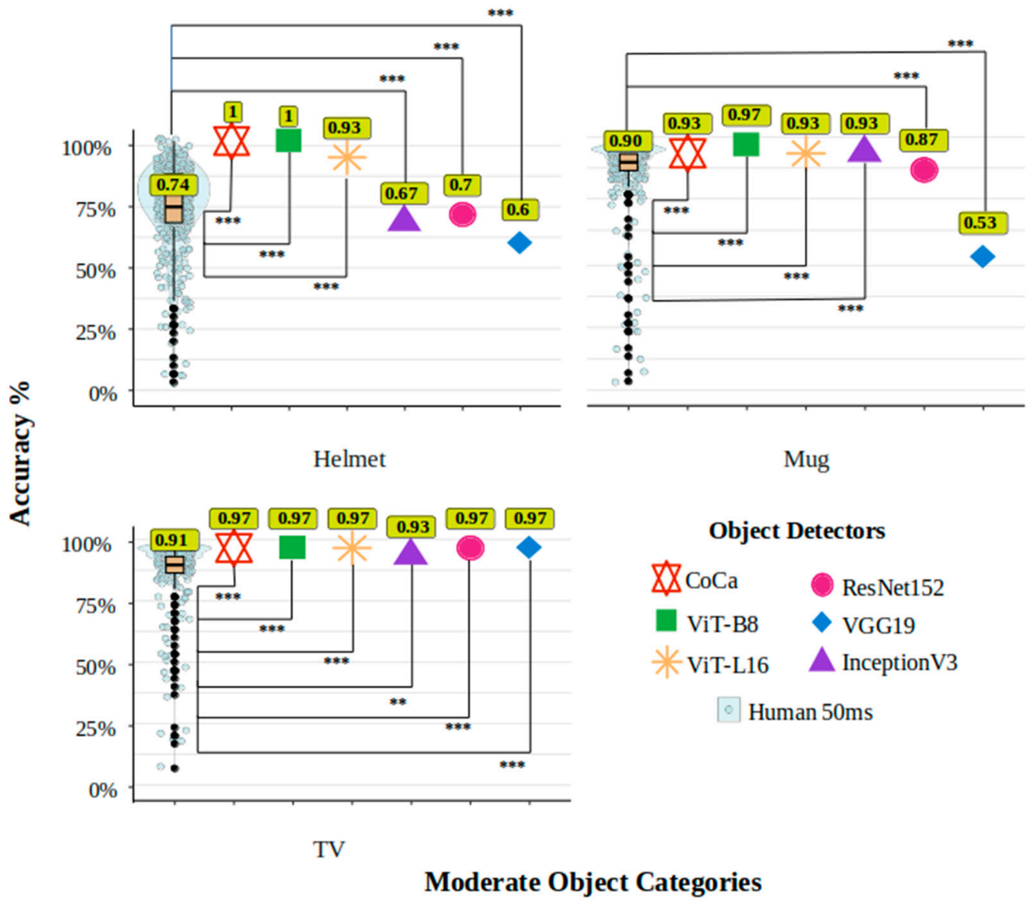

Figure S5. Accuracy of humans and the artificial vision systems by moderate object categories. "Human 50ms" denotes the accuracy of individuals during the test session at a 50ms presentation time.; CoCa, ViT-B8, ViT-L16, ResNet152, InceptionV3 and VGG19 are the artificial vision systems. The box plot showcases the mean performance and interquartile range of human data, with solid black circles indicating potential outliers identified using the interquartile range criterion. Significance lines indicate comparisons between human and model accuracies: advantages favouring humans are shown above the box plots, while those favouring each DNN are shown below. Different signifiers indicate varying levels of Bayesian evidence for differences, with * indicating BF > 3, ** indicating BF > 10, and *** indicating BF > 100.

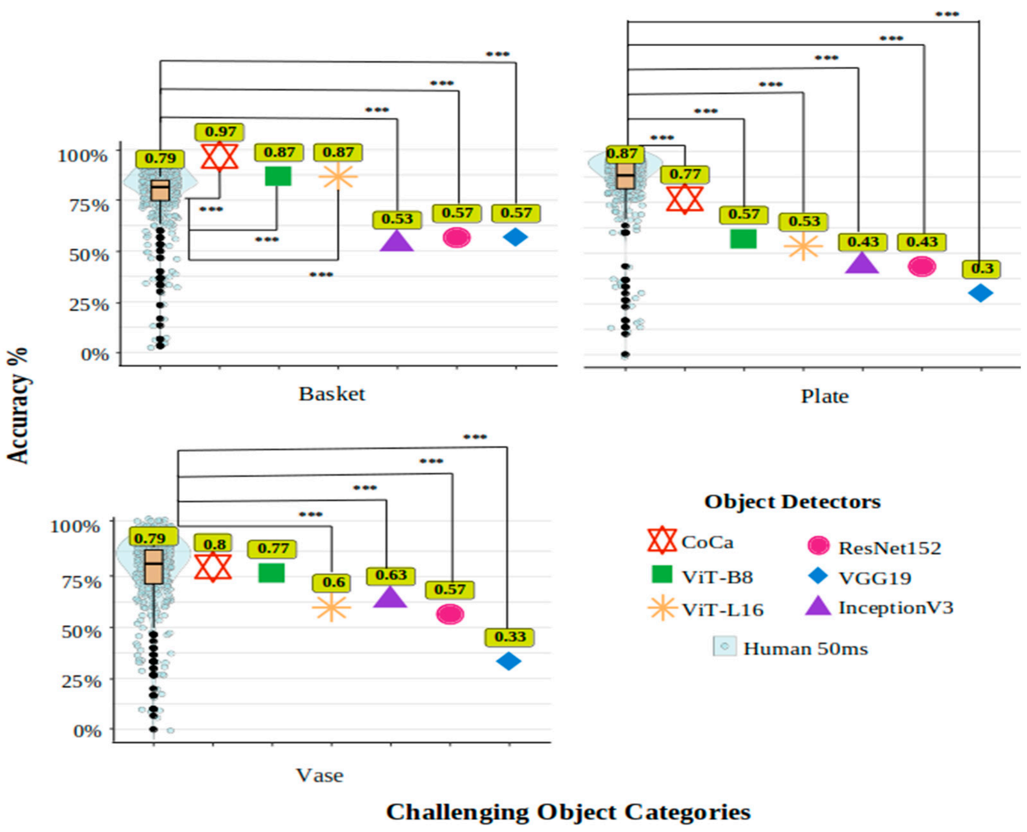

Figure S6. Accuracy of humans and artificial vision systems by challenging object categories. "Human 50ms" denotes the accuracy of individuals during the test session at a 50ms presentation time.; CoCa, ViT-B8, ViT-L16, ResNet152, InceptionV3 and VGG19 are the artificial vision systems. The box plot showcases the mean performance and interquartile range of human data, with solid black circles indicating potential outliers identified using the interquartile range criterion. Significance lines indicate comparisons between human and model accuracies: advantages favouring humans are shown above the box plots, while those favouring each DNN are shown below. Different signifiers indicate varying levels of Bayesian evidence for differences, with * indicating BF > 3, ** indicating BF > 10, and *** indicating BF > 100.
